# Sorbitol induces flavonoid accumulation as a secondary signal via the nanoencapsulated SPc/lncRNA809-MmNAC17 module against *Alternaria alternata* in *Malus micromalus*

**DOI:** 10.1186/s43897-024-00125-z

**Published:** 2025-01-31

**Authors:** Tingting Du, Dong Meng, Hongyan Cao, Yi Lian, Rui Wu, Tengyue Liu, Tianyi Wang, Cai Qin, Zhihua Song, Biying Dong, Yujie Fu, Qing Yang

**Affiliations:** Beijing Forestry University, Beijing, 100000 China

**Keywords:** Sorbitol, Flavonoids, Long non-coding RNA lncRNA809, Disease resistance of *Malus Micromalus*

## Abstract

Sorbitol is an important primary metabolite that serves as both a carbon source and signal to pathogens. The leaf diseases caused by *Alternata alternata* are particularly serious in crabapple (*Malus micromalus*). Here, we found that sorbitol can enhance the resistance of crabapple to *A. alternata* R1 by increasing the content of flavonoid catechin. Nanomaterials as an emerging technology tool can efficiently deliver lncRNA to target cells. Here, we found nanoencapsulated lncRNA809 (SPc/lncRNA809) exhibits significant resistance to R1strain. To elucidate the effect of SPc/lncRNA809 on flavonoids catechin synthesis, we observed the expression of lncRNA809 was consistent with that of *MmNAC17* which regulates the synthesis of catechin and both could jointly respond to sorbitol. MmNAC17 induced the accumulation of catechin *in vivo* by directly activating the expression of catechin synthase genes *MmF3H* and *MmLAR*. Correspondingly, overexpression of lncRNA809 significantly upregulated the expression of *MmNAC17* and enhanced the disease resistance. This study reveals for the first time that sorbitol positively regulates the expression of MmNAC17 through lncRNA809, promoting the accumulation of catechin via the expression of *MmF3H* and *MmLAR*, ultimately improving the defense response of *M. micromalus*. This research provides a crucial foundation for the establishment and application of sorbitol-based signaling regulatory networks.

**Supplementary Information:**

The online version contains supplementary material available at 10.1186/s43897-024-00125-z.

## Core

Sorbitol triggers *M. micromalus* with resistance to fungal infections by regulating the biosynthesis of catechin. The regulation of catechin biosynthesis by sorbitol is mainly achieved through the cascade reaction of lncRNA809-MmNAC17. Under pathogen infection, the transcription of lncRNA809 can be activated by the sorbitol signal. LncRNA809 positively regulates the expression of transcription factor MmNAC17, which in turn binds to the promoters of *MmF3H* and *MmLAR*, thereby positively regulating the biosynthesis of flavonoid catechins and ultimately enhancing the resistance of *M. micromalus* to *Alternaria* R1. At the same time, SPc nanomedicines derived from non-coding RNA lncRNA809 also have a good defense effect against *Alternaria* pathogens by mediating the biosynthesis of catechin.

## Gene & accession numbers

*LDOX-1XM_008358320.1*

*F3H-1NM_001293925.1*

*F3'H-6XM_008394995.2*

*CHS-2XM_029091251.1 ANS*

*KP742784.1 LAR-3NM_001293860.1*

*ERF4NM_001328880.1*

*NAC17HM122658.1 IBH1*

*XM_008367710.2 bZIP1LOC103406197*

## Introduction

In plant-microbe interactions, sugars produced by photosynthesis not only provide a carbon source for pathogens, but also fuel the growth and development of host plant. Sorbitol, a carbon reservoir of plants in the *Rosaceae* family, functions as the main accumulation of photosynthetic products. Increasing amounts of research have revealed that sorbitol also functions as a signaling molecule, contributing to plant defense against diseases. Aldose-6-phosphate reductase (A6PR) was identified as a key enzyme in sorbitol metabolism. In our previous research, two antisense A6PR lines were found to be susceptible to *A. alternata*, as the decrease of sorbitol altered the expression level of the disease resistant protein MdNLR16. When *MdNLR16* was overexpressed in two antisense A6PR lines, the plant was given enhanced resistance. Further, our data indicated that *MdWRKY79* functions as an upstream regulator of MdNLR16 in the signal transduction process in response to sorbitol, thus altering the resistance of apple leaves to *A.alternata *(Meng et al. 2018). Meanwhile, a reduction in sorbitol synthesis is associated with abnormal stamen development and diminished pollen tube growth in apple, mediated by the MYB transcription factor MdMYB39L. MdMYB39L binds to the promoter of*MdSTP13a*, a sugar transporter that imports both hexose and sucrose. This interaction activates MdSTP13a expression, which in turn influences pollen tube growth (Meng et al. 2018; Li et al. 2020). *Malus micromalus*, a plant in the *Rosaceae* family with ornamental, edible, and medicinal purposes, is widely distributed in northern China. Approximately 80% of this region is affected by fungal diseases of the genus *A.alternata*. In wet years, leaf spot-like symptoms were so severe that early defoliation occurred on these trees. Therefore, as an important economic forest species of the apple genus, it was also observed that external application of sorbitol exhibited minimal symptoms.

In the field of plant disease resistance research, diverse mechanisms of plant signal transduction have been identified, including transcriptional regulation (Qi et al. 2021). Long non-coding RNAs (lncRNAs; longer than 200 nucleotides) regulation of target gene expression (Palos et al. 2023), protein modifications including phosphorylation or ubiquitination and others (Zhang et al. 2023; Wang et al. 2023). It was reported the functional interpretation of long chain noncoding RNAs (lncRNAs; over 200 nucleotides) is challenging due to their low expression levels and a lack of sequence conservation. However, multiple types and origins of lncRNA have been identified and displayed in transcriptional regulation and chromatin modification (Kung et al. 2013; Laurent et al. 2015). It has been found that lncRNA SABC1 can act as a molecular switch to balance plant defense and growth by regulating SA biosynthesis (Liu et al. 2022). Upon activation of a plant’s disease resistance system, it promptly initiates a cascade of early immune responses, including calcium influx activation, phosphorylation of cytoplasmic kinases, as well as other signaling events. In addition, plant lncRNA plays a pivotal role in response to biotic and microbial stress (Tan 2020). Several lncRNAs that respond to pathogenic fungal infections have been identified in plants such as*Arabidopsis *(Zhu et al. 2014), wheat (Bilir et al. 2019), and tobacco (Zheng et al. 2021). LncRNA ELF18-INDUCED LONG-NONCODING RNA1 (ELENA1) has been reported to interact with the Mediator subunit 19a to regulate*PR1*, thereby enhancing resistance to *Pseudomonas syringe *(Seo et al. 2017). Moreover, it was reported that the first charaterization of resistance associated lncRNAs was conducted in two distinct cotton species, with one being resistant and the other susceptible to fungal disease (Zhang et al. 2018). Species-specific lncRNAs with a higher density of single nucleotide polymorphisms (SNPs) tend to exhibit more pronounced induction levels following infection compared to species-conserved (core) lncRNAs. These reports indicate that lncRNA plays an important role in the response of plants to fungal diseases.

In agricultural production, the prevention and control of economic forest diseases mainly depend on agricultural pesticides. However, the frequent use of pesticides has raised concerns about environmental impact, as well as human and ecological health and safety. Therefore, an alternative green technology should be developed to minimize toxicity, enhance target specificity, and ensure environmental sustainability. Excitingly, the emerging generation of advanced pesticides based on nanotechnology (nanopesticides) offers a highly efficient and low-toxicity solution for modern green prevention and treatment. As an emerging technology, nanomaterials can deliver siRNA, miRNA, and lncRNA to target cells, thereby achieving more efficient treatment. Here we constructed a facile-synthesized Star Polycation (SPc) as a low-cost dsRNA nanocarrier (Fig. 8A). The application of nanomaterials in plant science has significantly propelled the development of agriculture. The delivery of biomolecules (plasmid DNA, lncRNA, etc.) mediated by nanomaterials has achieved sustainable management of various diseases and pests. Consequently, nanocarriers may be an effective tool for enhancing plant immunity and inhibiting pathogens in the future.

Flavonoids, as an important secondary metabolite, are believed to play important roles in both biological and abiotic plants, serving as active oxygen scavengers. It was found in recent studies that lncRNAs regulate the synthesis of plant flavonoids by interacting with miRNAs and protein coding genes (PC genes) (Yang et al. 2019; Zhang et al. 2018). Activation of both PTI and ETI immediately initiate a series of early immune responses, including the activation of calcium influx, and the phosphorylation of cytoplasmic kinases and production of secondary metabolites (Lee et al. 2021). Research has shown that flavonoids in plants regulate drought and salt response by promoting antioxidant enzyme activity, and repairing damage caused by ultraviolet radiation. Abiotic stresses such as drought can also promote can also promote the synthesis and accumulation of flavonoids. It can be inferred that the biosynthesis and regulation of flavonoids are also closely related to biological stress. Specifically, flavonoids can resist microbial invasion in various ways, for example, by inhibiting bacterial toxin synthesis (Hertel et al. 2006), inhibiting bacterial migration (Rütschlin et al. 2020), inhibiting biofilm formation (Bjarnsholt et al. 2013), and inhibiting fungal hyphal growth (Zhang et al. 2022). Recently, it has been discovered that hormones like SA regulate the biosynthesis of catechin to resist rust in poplar trees (Rabiey et al. 2022). Catechin is readily induced and accumulated in antibacterial varieties of poplar. The antibacterial activity of catechin in vitro is regarded as considered one of the important reasons for the difference in resistance between antibacterial and non-antibacterial varieties of poplar, which has practical significance for tree breeding (Yang et al. 2023).

We know that calcium signaling is an important factor involved in plant defense research, and the changes in calcium ion influx can reflect the cell’s defense ability against fungal diseases. NMT (Non-invasive Micro-test Technology) is widely used in plant disease resistance research. NMT has emerged as a critical tool for monitoring calcium influx, but no studies to date have employed NMT technology to reflect the relationship between flavonoids and diseases resistance, especially in economic trees. In this study, NMT was used to measure calcium ions in leaf cells after fungal infection or sorbitol treatment. The instantaneous increase of intracellular calcium ions reflects the enhancement of resistance (Hao et al. 2024). The accumulation of flavonoids and its subsequent modulation of plant tolerance are subjects of increasing research interest. NMT is acknowledged for its convenience and clarity in reflecting the enhanced stress tolerance of cells.

In the era of genomics, gene regulatory networks (GRNs) have emerged as a prominent framework to understand the development and evolution of organisms. Therefore, researchers have conducted deep learning and established a multilayered hierarchical gene regulatory network centered around transcription factors (TFs), which helps plants cope with environmental fluctuations by dynamically regulating gene expression profiles. From another perspective, we believe that the expression of these core genes is guided by signals, that is, plants transmit information through the “signal waves” in their bodies. Therefore, this study mainly focuses on the signal hierarchy regulation relationship between primary and secondary metabolites in terms of signal transmission. In this work, we found that long non-coding RNA lncRNA809 is involved in regulating the expression of both *MmF3H* and *MmLAR*, which encode two key enzymes for catechin synthesis, in response to the primary signal sorbitol. Actually, lncRNA809 positively regulates the transcription of *MmNAC17*, which binds to the promoters of both *MmF3H* and *MmLAR* to activate their transcription. This process promote the accumulation of catechin (the secondary signaling molecule) thereby enhancing the disease resistance of *Malus micromalus*.

## Results

### The primary signaling molecule, sorbitol, significantly enhanced the resistance of *Malus micromalus* to the *A. alternata* R1 strain

**Fig. 1:**
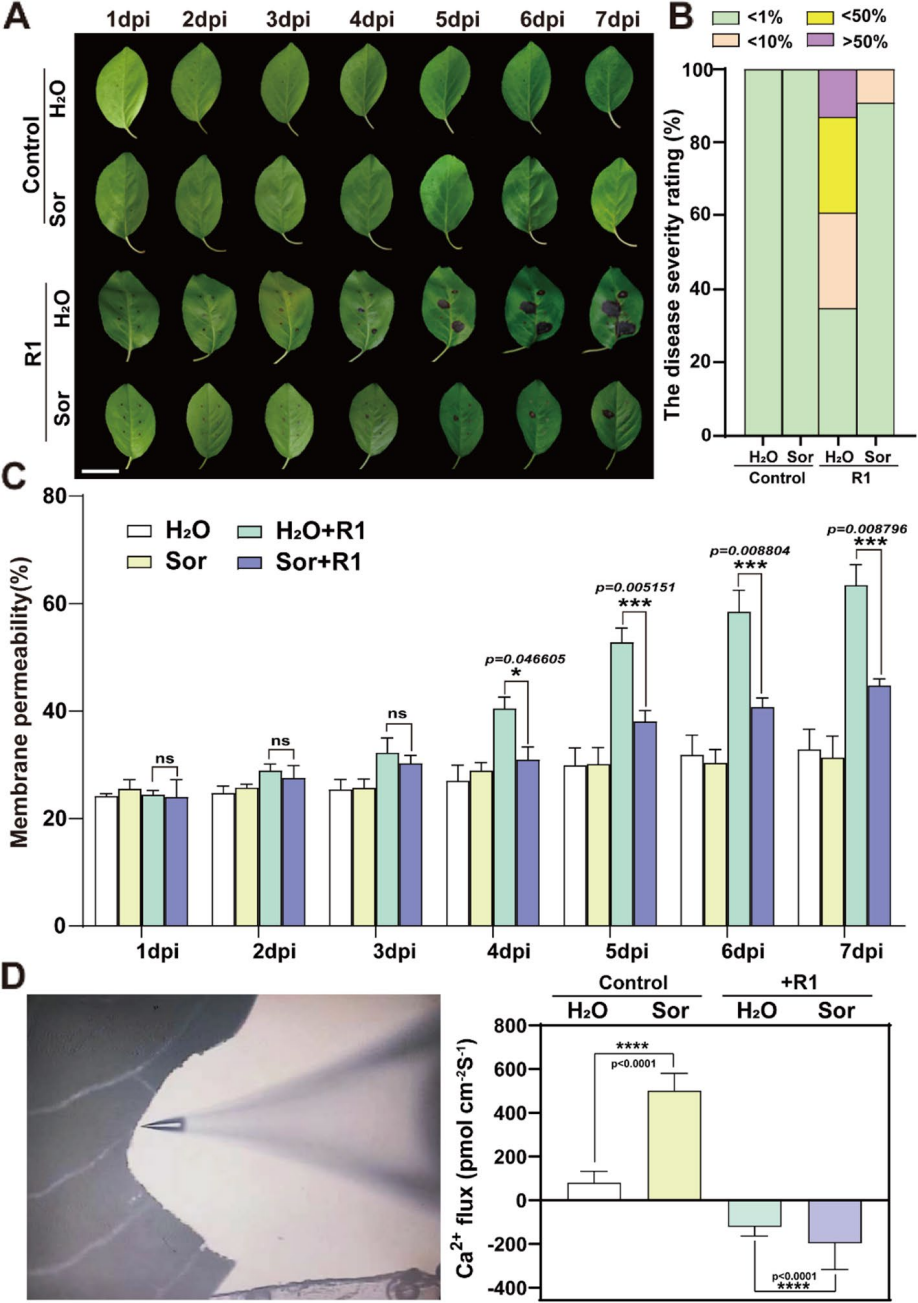
Sorbitol enhances the resistance of *M. micromalus * to pathogenic fungus R1. **A-C** leaves that have not been treated with sorbitol are more susceptible to *Alternaria* R1 infection. Young leaves were placed in 50 mM sorbitol solution and pure water for 6 h, and conidiospores of R1 were drop-inoculated onto each leaf and kept in a high humidity environment for 7 days. The representative phenotypes **A**, plant disease severity **B**, and cell membrane permeability (**C**) of R1-inoculated leaves are shown. The disease severity rating in (**B**) is represented as healthy (< 1%), mild symptoms (< 10%), moderate symptoms (10 -50%), and severe symptoms (> 50%). 40–50 leaves are used for the statistics. Here, 1%,10% and 50% represent the percentage of lesion area to leaf area. Different colors represent different rating. Ordinate represents percentage of different levels. **D** Sorbitol can induce early Ca^2+^ signaling. Detached leaves were drop-inoculated with conidiospores of R1 for 4 days. NMT technology was used to detect Ca^2+^ flux. The instantaneous Ca^2+^ flux after adding sorbitol to the test solution reflects the effect of sorbitol. The steady-state fluxes were continuously recorded for 5 min. The data are the mean ± SD. *p < 0.05, ***p < 0.001, ****p < 0.0001, two-sided Student’s t-test. Scale bars, 4.5 cm

Sorbitol is the main photosynthetic product of plants in the *Rosaceae* family. Previous studies have found that reduced synthesis of sorbitol leads to downregulation of the *MdNLR*gene, thereby reducing the resistance of apple seedlings to pathogenic fungi (Dong Meng et al. 2018). *M. micromalus* belongs to the apple genus that has a similar photosynthetic product to apples—sorbitol. To determine whether sorbitol in *M. micromalus* exhibits disease resistance, a series of sorbitol feeding experiments were conducted. Firstly, we isolated three pathogenic fungi from the diseased leaves, named “R1”, “R2”, and “R3”, respectively (Supplemental Fig. 1A). The three pathogenic fungi were re-inoculated onto the leaves to verify their infectivity, and the results showed that R1 has strong pathogenicity (Supplemental Fig. 1B). Therefore, R1 is the main pathogenic fungus causing the brown spot disease of *M. micromalus*. Sequence identification and phylogenetic analysis confirmed that R1 belongs to the *A. alternata* R1 fungi (Supplemental Fig. 1C). Subsequently, it was verified that 50 mM sorbitol had a significant inhibitory effect on *A. alternata* R1. Sorbitol was used to continuously feed the leaves of *M. micromalus* for 6 h. After that, the pathogen inoculation was carried out on the surface of the leaves, and leaf lesions were subsequently observed following infection. We fed leaves only with water as control. Exogenous feeding of sorbitol can increase the endogenous content of sorbitol in leaves (Supplemental Fig. 2). The proportion of disease spots formed on leaves that were fed with sorbitol and R1-infected was higher than that of the control group without inoculation, but significantly lower than that of leaves that were not fed with sorbitol but infected with R1. This indicates that leaves treated with sorbitol showed better resistance. On the 7th day after inoculation, 26% of the leaves infected with R1 but not fed with sorbitol were mildly infected (lesion area < 10%), and 26% were moderately infected (lesion area 10 -50%). In contrast, leaves fed with sorbitol had a lesion area of less than 10% (Fig. 1A-B). To observe the development of lesions more vividly, we created movies based on the continuous phenotypes of four treated leaves. The fungal infection can be clearly seen (movies1-4). To describe the extent of leaf damage more specifically, cell membrane permeability was used as a key indicator in different treatment groups. The cell membrane permeability of the leaves in the sorbitol fed group and R1-inoculated group was significantly lower than that of the only inoculated group from 4th day, indicating that sorbitol can alleviate the degree of disease infection to a certain extent (Fig. 1C). In order to further illustrate the conclusion that sorbitol can alleviate fungal disease invasion, we chose Ca^2+^ as an important indicator. Ca^2+^ is activated in the early stages of pathogen invasion. The rapid increase in Ca^2+^concentration will trigger downstream signal transduction, leading to plant immune responses (Hao et al. 2024). To some extent, we can use Ca^2+^ influx to represent the enhanced disease resistance of *M. micromalus* leaves due to sorbitol. To further prove our hypothesis, the Ca^2+^ fluxes of the infected leaves after sorbitol exposure were measured by Noninvasive Microtest Technology (NMT). The Ca^2+^ in untreated mesophyll cells was in a slight efflux state (about 80 pmol cm^−1^ sec^−1^), while the sorbitol treatment significantly increased Ca^2+^ efflux, with a Ca^2+^ flux value of 500 pmol cm^−1^ sec^−1^. Pathogen infection significantly increased intracellular Ca^2+^ fluxes relative to the healthy control: Ca^2+^ influx induced by R1 increased by 201.5 pmol cm^−1^ sec^−1^ in leaves not fed with sorbitol, and the intracellular Ca^2+^ level reached about − 121.5 pmol cm^−1^ sec^−1^. However, Ca^2+^ influx induced by R1 increased by 696 pmol cm^−1^ sec^−1^ in leaves fed with sorbitol, and the Ca^2+^ flux value ultimately reached about − 196 pmol cm^−1^ sec^−1^ (Fig. 1D). Since sorbitol treatment led to a stronger calcium influx, so we believe that sorbitol can enhance the resistance of *M. micromalus* to *A. alternata* R1.

### Sorbitol activating the expression of genes involved in flavonoid biosynthesis for plant defense

**Fig. 2:**
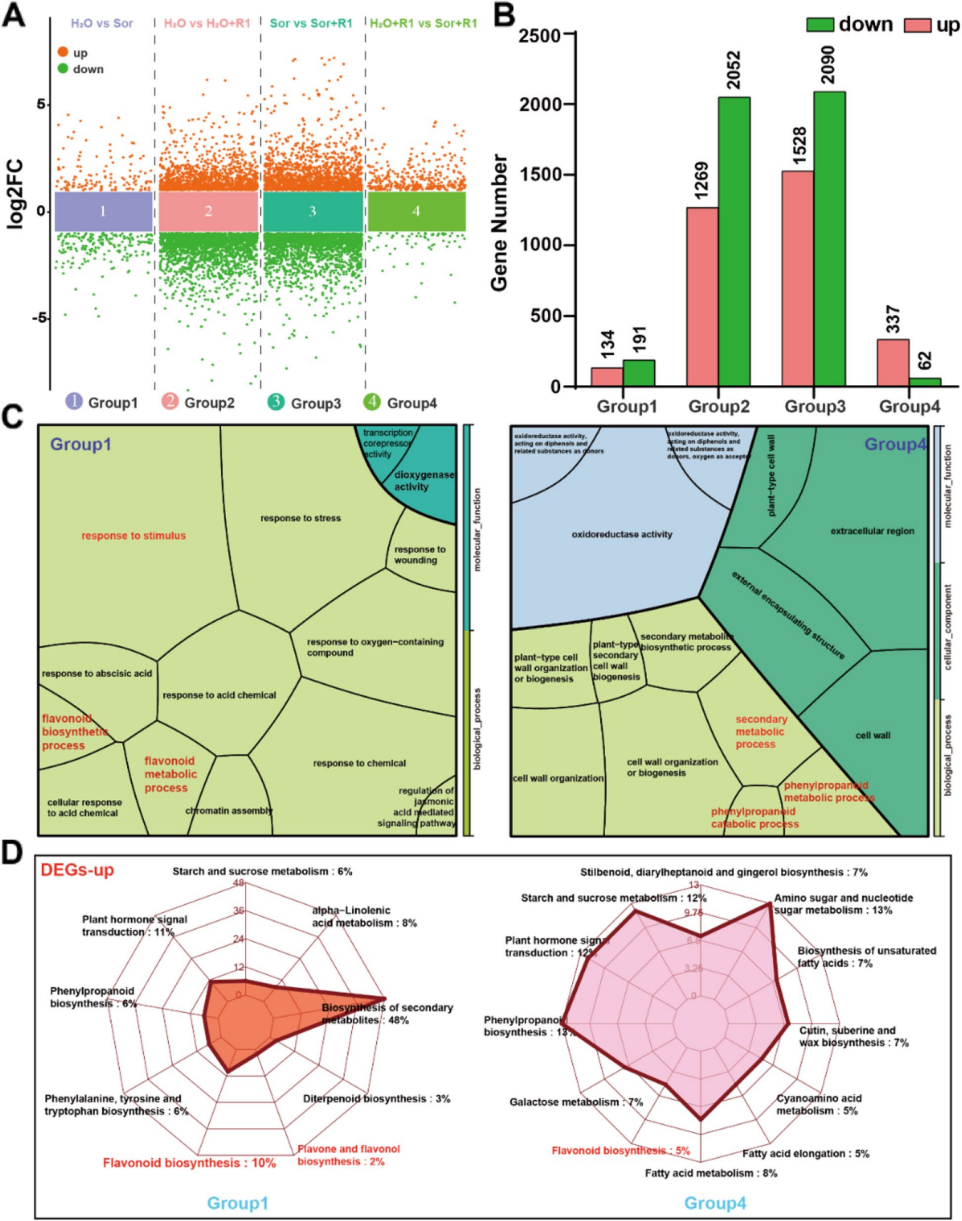
Transcriptome data analysis of leaves fed with water, fed with sorbitol, inoculated with R1, and inoculated with R1 after feeding with sorbitol. **A** Volcano plot of differentially expressed genes in Group 1 (H_2_O vs. sorbitol), Group 2 (H_2_O vs. H_2_O + R1), Group 3 (sorbitol vs. sorbitol + R1), Group 4 (H_2_O + R1 vs. sorbitol + R1). **B** Statistical analysis of up-regulated and down-regulated genes in four comparison groups. **C** GO enrichment analysis of differentially expressed genes in Group1 and Group4. Here are the top 15 entries with *p* < 0.05. **D** KEGG enrichment analysis of differentially upregulated genes in Group 1 and Group 4

Our previous research has indicated that sorbitol acts as a function as a primary signaling molecule in the resistance and development of apples and loquats (Meng et al. 2018; Li et al. 2020; Xu et al. 2023). Therefore, it is essential to investigate which metabolic pathways and key genes are regulated by sorbitol to jointly promote the disease resistance of*M. micromalus*. We performed transcriptome sequencing on four groups of treated leaves infected with R1 for 7 days: two groups of healthy control leaves (treated with water and sorbitol respectively), and two groups of leaves inoculated with R1 (infected with water/R1 and sorbitol/R1 respectively). We constructed 12 cDNA libraries and obtained 69.931 Gb of clean data. The average Clean Data of each group of samples was 5.828Gb, and Q30 base percentage was above 91.89%. The expression levels of 34,388 known genes were detected. We first conducted an intergroup differential expression analysis of these genes and categorized the pairwise comparison among different treatment groups into four distinct groups. Group 1 represents the comparison between sorbitol and water. Group 2 represents the comparison between R1 infection and the water control group. Group 3 represents the comparison between post-sorbitol feeding R1 infection and sorbitol control group. Group 4 represents the comparison between R1 infection after sorbitol feeding and R1 infection after water feeding. The volcano plot reveals that the differential genes between Group 2 and Group3 reflect the significant impact of pathogenic fungal infection on the entire transcriptional level, while the treatment with sorbitol (Group 1 and Group 4) may have a certain effect (Fig. 2A). Specifically, fungal infection led to significantly more downregulated genes than upregulated genes. Group 2 and Group 3 had 2052 and 2090 downregulated differentially expressed genes (DEGs), respectively. However, Group 3 had more DEGs than Group 2, which indicates that feeding with sorbitol increased the number of DEGs. Group 4 revealed that sorbitol promoted differential upregulation of 337 genes and downregulation of 62 genes (Fig. 2B). In summary, the DEGs among different groups reflected the role of sorbitol in plant disease resistance by altering gene transcription levels.

We wish to further explore which secondary metabolic pathways are regulated by sorbitol to take part in plant disease resistance responses. Therefore, we performed GO functional enrichment and KEGG pathway analysis on four sets of differentially expressed genes. In Group 1, 97 GO entries were significantly enriched. Among them, biological processes such as “flavonoid biosynthetic process”, “flavonoid metabolic process”, and “response to stimulus” were highly representative. In Group 4, 123 GO entries were significantly enriched. We focused on the top three biological processes, namely “cell wall organization or biogenesis”, “secondary metabolic process”, and “phenylpropanoid metabolic process”. The results of Group 1 and Group 4 suggest that sorbitol may enhance leaf disease resistance through flavonoid metabolism (Fig. 2C). We also analyzed the metabolic pathways enriched by DEGs in Group 2 and Group 3, and identified that “flavonoid biosynthetic process” and “flavonoid metabolic process” were significantly enriched under R1 infection (Supplemental Fig. 3). This observation not only indicates the potential role of flavonoids in resisting pathogenic fungi but also provides a basis for understanding how flavonoid metabolic pathways mediated by sorbitol contribute to plant defense responses.

Therefore, we continued to focus on the biological processes enriched by the differentially expressed genes of the four groups mentioned above. We also conducted detailed KEGG analysis on the upregulated and downregulated genes, respectively, to confirm the potential role of flavonoid biosynthesis in pathogenic fungal infection. In particular, differentially expressed genes (DEGs) that are significantly enriched in the “flavonoid biosynthesis” and “flavone and flavonol biosynthesis” pathways were upregulated in Group 1 and Group 4 (Fig. 2D and Supplemental Fig. 4A). In contrast, these genes were significantly downregulated under pathogen infection in Group 2 and Group 3 (Supplemental Fig. 4B). Therefore, we believe that sorbitol mainly triggers plant resistance to pathogen invasion by altering the transcription levels of genes involved in flavonoid synthesis, which also indicates the important role of flavonoid biosynthesis in plant defense.

### The secondary signaling flavonoid substance catechin was induced by sorbitol to enhance defense against *A. alternata* R1 strain

**Fig. 3:**
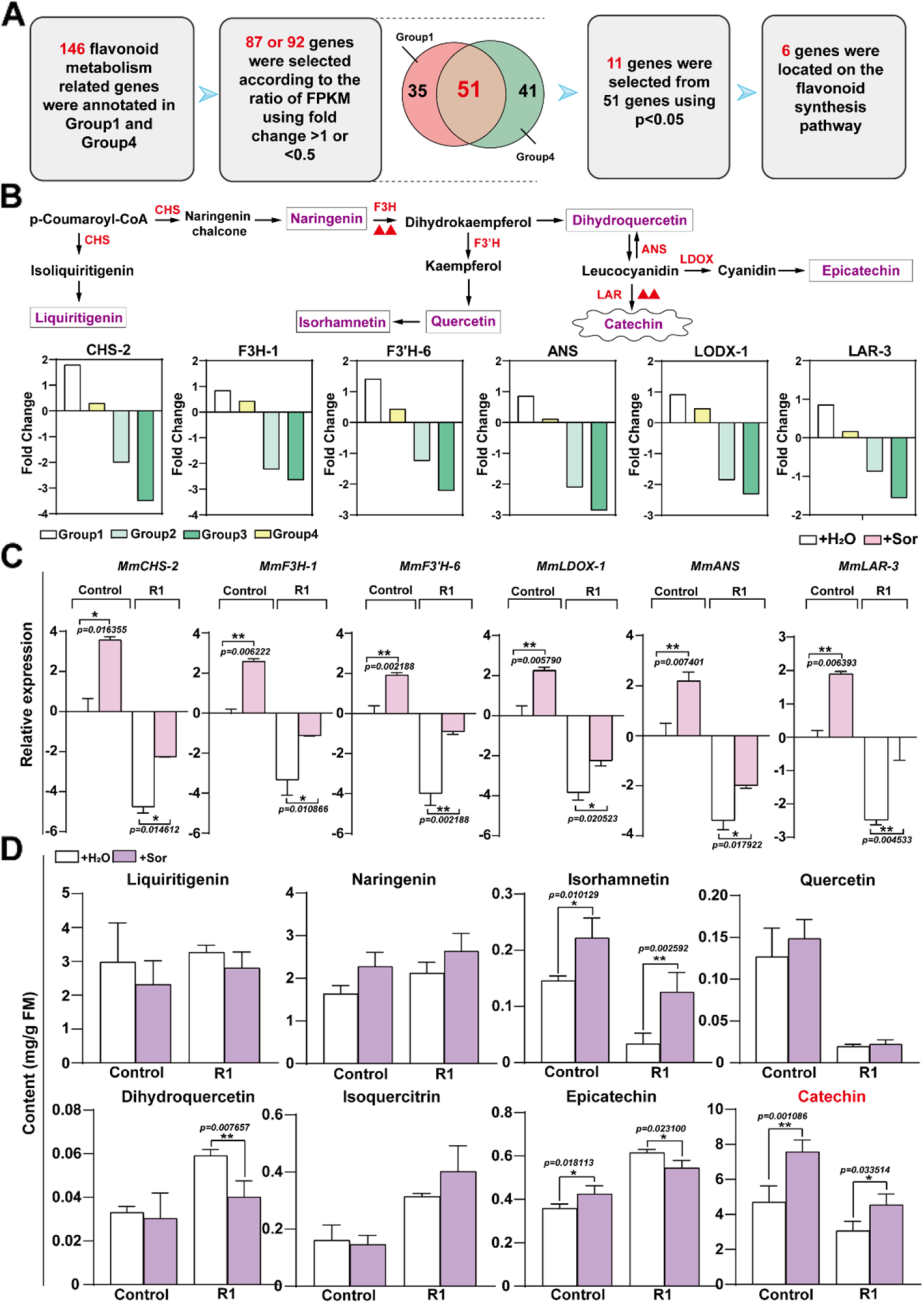
Sorbitol significantly increases the content of catechin, a flavonoid metabolite in the leaves to cope with R1 fungal infection. **A** Screening of genes involved in flavonoid metabolism pathways. The differential genes enriched in flavonoid metabolism pathways in Group 1 and Group 4 were integrated, with a fold change ≥ 1 or ≤ 0.5, p-value ≤ 0.05. **B** Display of the location of differentially expressed genes screened in the flavonoid metabolism pathway. The bar chart represents the expression levels of genes based on transcriptome data. The red triangle represents the genes that will be the focus of the following text. **C** RT-qPCR analysis of the genes under sorbitol feeding and pathogen infection. **D** Identification of differential metabolites under sorbitol feeding and pathogen infection. For C and D, a two-tailed Student’s t-test was used (mean ± SD; *n* = 5)

As shown in previous research results, feeding with sorbitol remarkably enhances the tolerance of *M. micromalus* leaves against pathogenic fungi (Fig. 1). The KEGG metabolite pathway enrichment analysis showed that sorbitol feeding had a significant impact on the flavonoid biosynthesis process. It is necessary to clarify the relationship between sorbitol and flavonoid, as well as to understand the role of flavonoids in resisting pathogenic fungi. The omics analysis results indicated that the flavonoid synthesis-related genes were predominantly upregulated rather than downregulated after sorbitol treatment (Group 1). Upon *A. alternata* R1 infection, the number of upregulated genes related to flavonoid synthesis in the leaves pre-treated with sorbitol significantly increased compared to those pretreated with the water (Supplemental Fig. 5). Consequently, we analyzed 146 flavonoid synthesis-related genes responsive to sorbitol. Among these, 87 genes were differentially expressed after sorbitol feeding (Group 1, fold change > 1 or < 0.5), and 92 genes were differentially expressed after inoculation with *A. alternata* R1 following sorbitol feeding (Group 4). An overlap of 51 genes was observed between the two groups, indicating shared regulatory responses to sorbitol. Based on a selection strategy of p < 0.05, we identified 11 significantly up-regulated candidate genes from the 51 genes. Among these, 6 genes were annotated within the flavonoid biosynthesis pathway (Fig. 3A), encoding CHS (Chalcone Synthase), F3H (Flavonoid 3-hydroxylase), F3’H (Flavonoid 3’-hydroxylase), ANS (Anthocyanidin Synthase), LDOX (Leucoanthocyanidin O-methyltransferase), and LAR (Leucoanthocyanidin Reductase), respectively. These genes were mainly involved in the conversion process of p-Coumaroyl-CoA to final products such as isorhamnetin, catechin, and epicatechin (Fig. 3B).

We also analyzed the expression levels of other members of these gene families and discovered that they either did not show a significant response to sorbitol or did not show significant response to *A.alternata* R1 (Supplemental Fig. 6). To demonstrate the relationship between sorbitol and flavonoid, we observed that after sorbitol feeding, the expression levels of these flavonoid synthase genes increased and then decreased after pathogen infection, which was consistent with the transcriptome data (Fig. 3C). Then, we focused on the accumulation of the eight important typical flavonoids including liquiritigenin, naringin, dihydroquercetin, quercetin, isorhamnetin, isoquercitrin, epicatechin, and catechin, which correspond to the six flavonoid synthases genes after sorbitol feeding. High performance liquid chromatography (HPLC) detection analysis revealed that the levels of naringin, isorhamnetin, and catechin in the leaves of the sorbitol-fed group were higher than those in the water-fed group, and these levels remained at higher levels even after pathogen infection (Fig. 3D). In particular, catechin exhibited a notably higher content and displayed significant variability, which is the focus of our subsequent investigation.

**Fig. 4:**
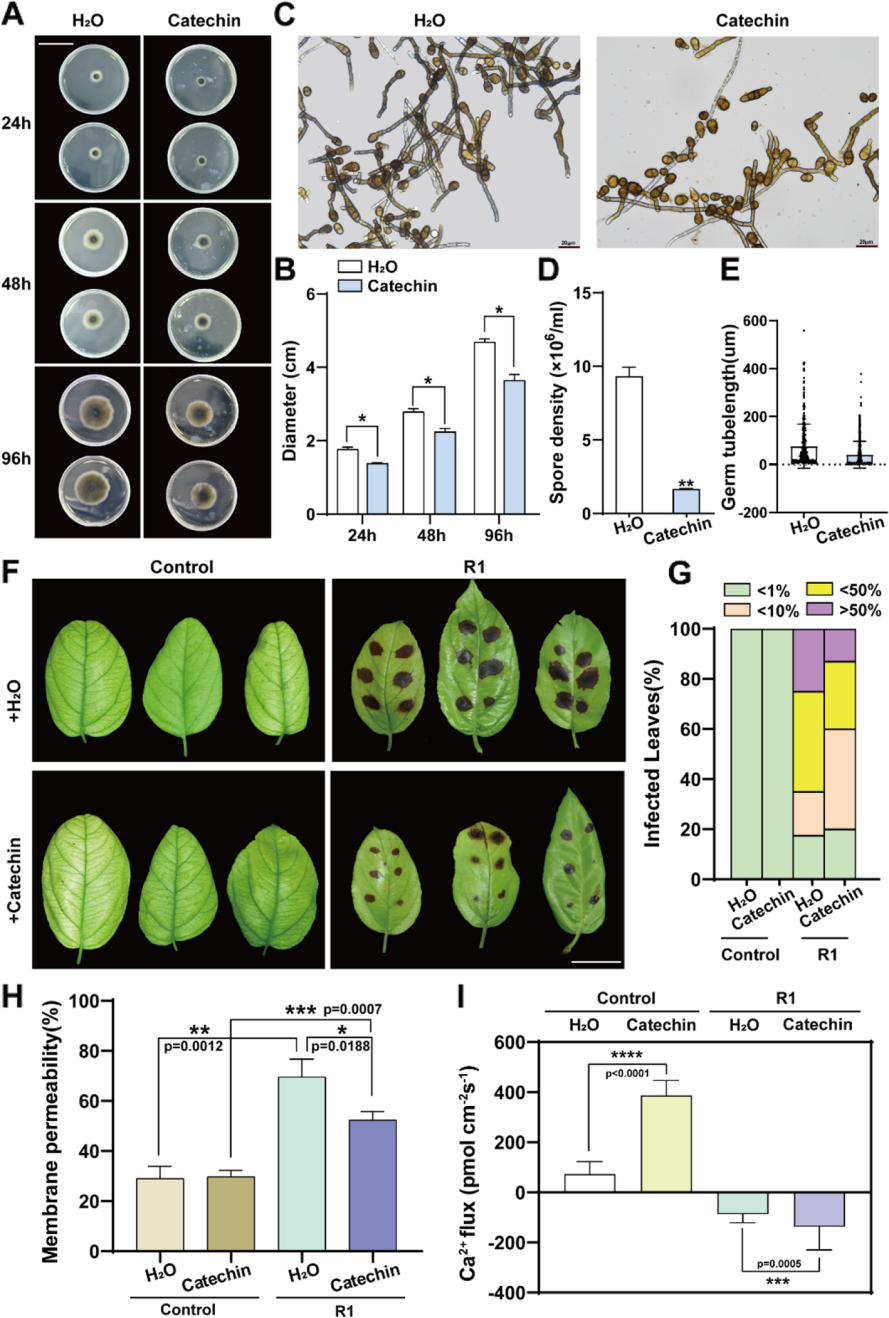
Flavonoids metabolite catechin can inhibit the growth of pathogens and enhance the resistance of leaves to R1. **A**-**E** Catechin inhibits the growth of pathogenic fungus R1 in vitro. R1 was inoculated onto PDA medium with catechin and cultured at 25℃ for 96 h. The growth process (**A**) and spore morphology (**C**) of pathogen R1 are displayed in the presence or absence of catechin. Scale bars, 4.5 cm (**A**) and 20 μm (**C**). The growth diameter (**B**), spore quantity (**D**), and spore germination (**E**) are also statistically analyzed. (**F**-**I**) Catechin enhances the resistance of *M. micromalus* leaves to R1. Catechin was applied to the surface of detached leaves for 6 h, and conidiospores of R1 were drop-inoculated onto each leaf and kept in a high humidity environment for 7 days. The representative phenotypes (**F**), plant disease severity (**G**), and cell membrane permeability (**H**) of R1-inoculated leaves are shown. The disease severity rating in (**G**) is represented as healthy (< 1%), mild symptoms (< 10%), moderate symptoms (10 -50%), and severe symptoms (> 50%). **I** Catechin can induce early Ca^2+^ signaling. Detached leaves were drop-inoculated with conidiospores of R1 for 4 days. NMT technology was used to detect Ca^2+^ flux. The instantaneous Ca^2+^ flux after adding catechin to the test solution reflects the effect of catechin. The steady-state fluxes were continuously recorded for 5 min. Scale bar, 2 cm. The data are the mean ± SD. *p < 0.05, **p < 0.01, ***p < 0.001, ****p < 0.0001, two-sided Student’s t-test

In order to investigate whether catechin has inhibitory effects on *A. alternata* R1, we first set up four catechin standard solutions with different concentration gradients and added them to PDA medium to determine the optimal inhibitory concentration. The results showed that as the concentration of catechin increased, the growth rate of the fungi decreased (Supplemental Fig. 7A). The growth diameter of *A. alternata* R1 slowed down with the increase in catechin concentration, and the number of their spores also decreased as the concentration of catechin rose (Supplemental Fig. 7B-C). 10 mg/L and 20 mg/L catechin were found to exhibit the most obvious inhibitory effect on *A. alternata* R1, with similar antifungal efficacy. Consequently, we selected 10 mg/L catechin for the subsequent experiments. Following 24 h of co-cultivation, the growth of the fungal strain *A. alternata* R1 was significantly impeded by the presence of 10 mg/L catechin, with the inhibitory effect becoming more pronounced over time. After 96 h of co-cultivation, the expansion diameter of *A. alternata* R1 had reached 4.7 cm in the culture medium without catechin, while the expansion diameter was 3.6 cm in the medium with catechin (Fig. 4A-B). We also observed the growth of spores under a microscope (Fig. 4C) and measured the number of fungal spores and the length of germ tubes. The data revealed that catechin significantly reduced both the number of fungal spores and the germination rate (Fig. 4D-E). In addition, we also focused on the antifungal effects of other flavonoids, and it was found that these flavonoids exerted a certain degree of inhibition on *A. alternata* R1. Compared with the control group, naringin, quercetin, isorhamnetin, isoquercitrin, dihydroquercetin, and epicatechin showed a significant inhibitory effect on the growth diameter of *A. alternata* R1 after 48 h of co-cultivation, however, the inhibitory effect weakened at 96 h. Moreover, the spore density and the length of germ tubes were significantly reduced by these flavonoids, although none of them showed the same inhibitory potency as catechin (Supplemental Figs. 8–13). These results indicate that flavonoids have a significant inhibitory effect on fungi, albeit with specificity and selectivity across different species. Specifically, catechin plays a pivotal role in disease resistance in the leaves of Begonia, and it was the sole flavonoid strongly induced by sorbitol. We further explored whether catechin could enhance the plant disease resistance. We collected *M. micromalus* leaves and applied catechin to the surface of the leaves for 6 h before inoculation with *A. alternata* R1. The leaves treated with catechin exhibited significantly smaller lesion areas compared to the control (Fig. 4F). According to the statistical data, approximately 60% of the leaves that were pre-treated with catechin were mildly infected with *A. alternata* R1, around 27% were moderately infected, and about 13% were severely infected. In control group, approximately 35% had mild infection, 40% with moderate infection, and 25% with severe infection (Fig. 4G). In addition, the leaves treated with catechin also showed lower cell membrane permeability after *A. alternata* R1 infection (Fig. 4H), indicating that catechin can alleviate the degree of disease infection. Our previous research has shown that intracellular calcium sensors such as CDPK, CIPK14, and CBL1 can directly or indirectly regulate the expression of genes encoding flavonoid biosynthesis enzymes, and alter flavonoid biosynthesis level to resist stress (Yang et al. 2020; Meng et al. 2021). A mysterious association is shown between calcium signal and flavonoids. We wanted to know if flavonoids would cause changes in intracellular calcium ion levels, and we conducted NMT analysis on the Ca^2+^ fluxes of infected leaves pretreated with catechin, and the results showed that when the leaves were not infected with R1, catechin significantly increased Ca^2+^ fluxes. After *A. alternata R1* infection, the intracellular Ca^2+^ fluxes of catechin treated leaves reached to -137 pmol cm^−1^ sec^−1^, while the intracellular Ca^2+^ fluxes of the water treatment group was about − 87 pmol cm^−1^ sec^−1^ (Fig. 4I). Clearly, catechin treatment also led to stronger calcium influx, which is similar effect observed with sorbitol. The above results further support the viewpoint that sorbitol regulates the biosynthesis of catechin to resist diseases.

### The expression of transcription factor *MmNAC17* was induced by sorbitol and enhanced resistance to *A. alternata* R1

**Fig. 5:**
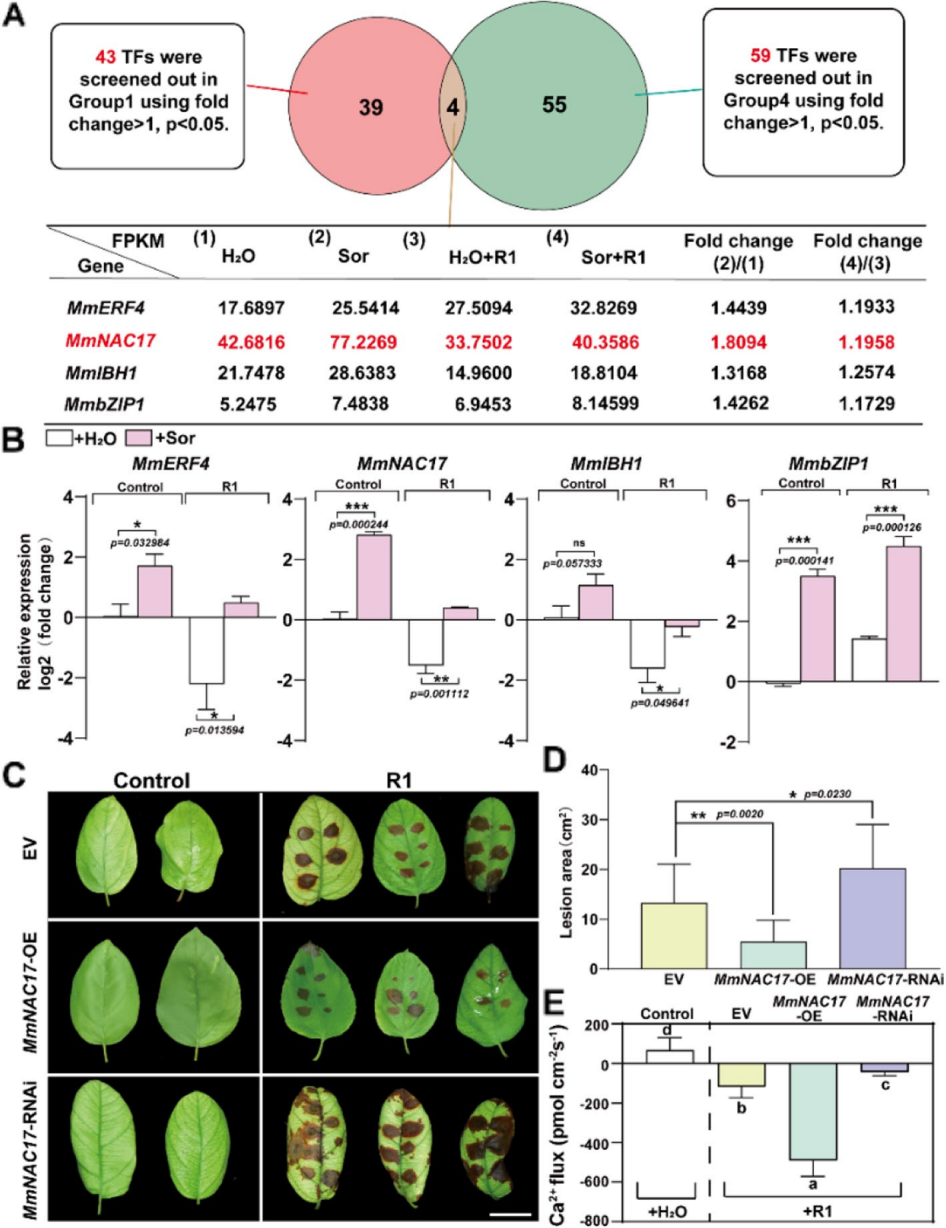
Sorbitol enhances the resistance of *M. micromalus * to R1 by inducing transcription factor MmNAC17 expression. **A** Screening for differentially upregulated transcription factors. The differential transcription factors in Group 1 and Group 4 were integrated, and there were four genes with a fold change ≥ 1, P-value ≤ 0.05. **B** Relative expression level of transcription factors in leaves under sorbitol feeding and pathogen R1 infection. Transcripts were quantified by RT-qPCR. The data is the mean ± SD (*n* = 3). *P < 0.05, **p < 0.01, ***p < 0.001, ns, not significant, two-sided Student’s t-test. **C**-**D** MmNAC17 regulates the disease resistance of leaves. Transgenic *M. micromalus* leaves were inoculated with R1 for 5 days. The representative phenotypes (**C**), quantification data of pathogen invasion severity (**D**) and Ca^2+^ flux (**E**) are shown. ANOVA, Tukey’s multiple comparisons, *P* < 0.05, different letters indicate significant differences, Scale bar, 2 cm

**Fig. 6:**
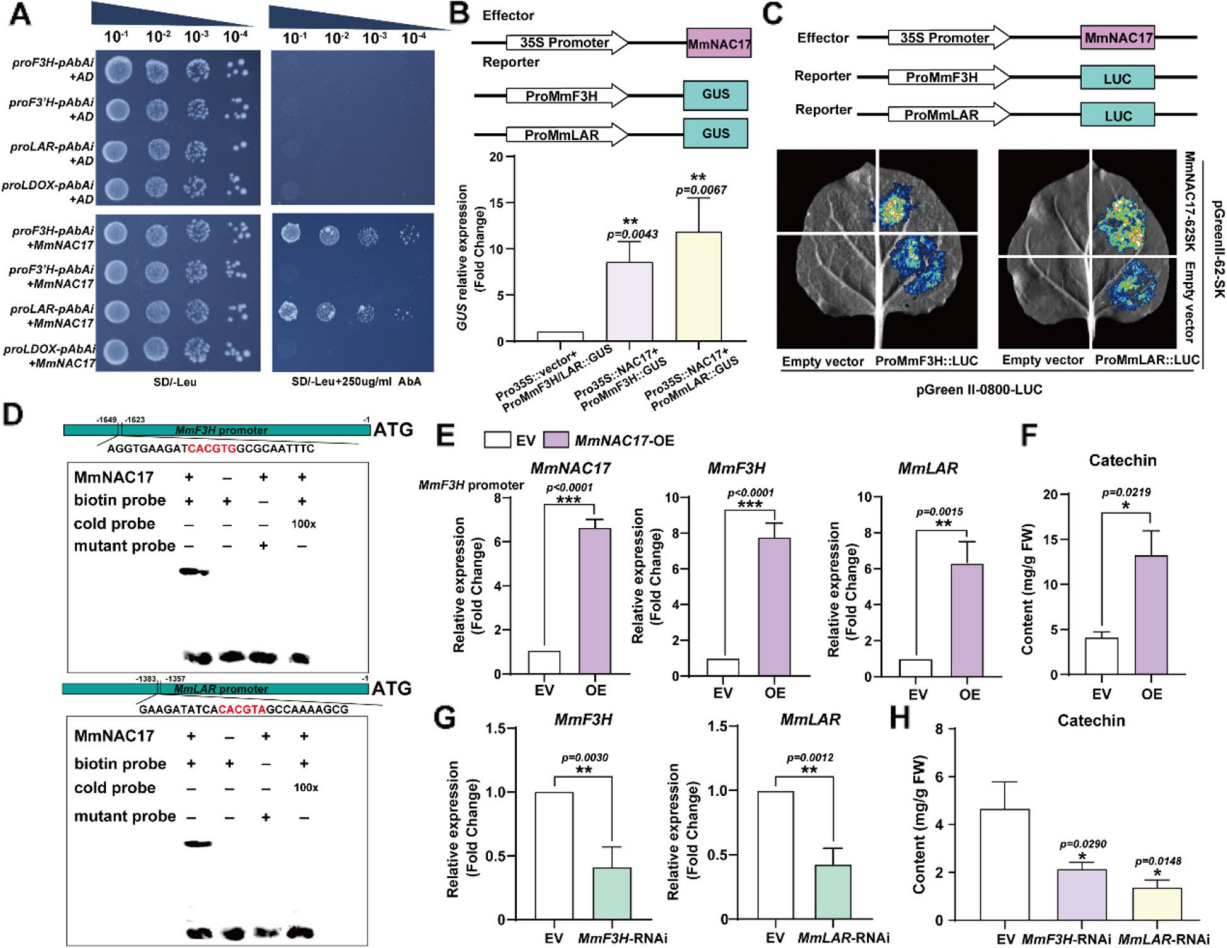
MmNAC17 simultaneously binds to the promoters of*F3H* and *LAR* and promotes their expression, thereby promoting the accumulation of catechin. **A** Y1H assays showing the interaction between MmNAC17 and F3H and LAR promoter fragments. Empty pGADT7-Rec2 vectors were used as a negative control. AbA: Aureobasidin A. **B** The GUS expression system indicates that MmNAC17 can bind to the promoters of F3H and LAR in vivo. **C** The luciferase assay showed that MmNAC17 activated the *MmF3H* and *MmLAR* promoters in *N. benthamiana* leaves. 35 S::MmNAC17 co-expressed with *promoter: LUC*, and negative control of 35 S::MmNAC17 or *promoter: LUC* was indicated, respectively. **D** EMSA indicating the binding of MmNAC17 to the *MmF3H* and *MmLAR* promoters in vitro. A schematic of the position in the promoter was shown. A 26 bp DNA fragment containing “CACGTG” and “CACGTA” was used, respectively. “+” and “-” indicate the presence and absence of the corresponding proteins and probes, respectively. 100× diluted unlabeled probes were added as competing probes. **E** and **G** Relative expression levels of F3H and LAR in instantaneous transgenic leaves. Transcripts were quantified by RT-qPCR. **F** and **H** The content of catechin in leaves overexpressing MmNAC17 and inhibiting the expression of F3H and LAR, respectively. Catechin was quantified by HPLC. The data is the mean ± SD (*n* = 3). *P < 0.05, **p < 0.01, ***p < 0.001, two-sided Student’s t-test

To identify transcription factors that may regulate catechin biosynthesis and disease resistance, we conducted a comprehensive analysis of all transcription factors responsive to sorbitol using transcriptome data. It was found that the number of up-regulated transcription factors was less than that of down-regulated genes in the late stages of pathogen infection (in Groups 2 and 3). However, due to the promotion of gene expression upregulation by sorbitol treatment (Group1), the number of downregulated transcription factors decreased, and the number of upregulated transcription factors increased in leaves pretreated with sorbitol under R1 infection compared to the control group (Supplemental Fig. 14). Given that the expression of transcription factors and flavonoid synthase genes is quite consistent, we believe that there must be a transcription factor that regulates the expression of flavonoid synthase genes to enhance the resistance of *M. micromalus*. We identified four transcription factors that were differentially upregulated between Groups 1 and 4 (Fig. 5A), including *MmERF4*, *MmNAC17*, *MmIBH1*, and *MmbZIP1*, which were all significantly upregulated in response to sorbitol, with *MmNAC17* exhibiting a particularly high degree of consistency in expression with the flavonoid catechin content pattern (Fig. 5B and D). Therefore, *MmNAC17* may play a more important role in catechin biosynthesis and resistance to fungal diseases caused by *A. alternata* R1. To verify the function of MmNAC17 under pathogen infection in leaves, we determined disease tolerance using *MmNAC17*-RNAi and *MmNAC17*-OE plants and compared their leaves phenotypes with the empty control. Before inoculating with *A. alternata* R1, the transcript level of *MmNAC17* was first monitored in *MmNAC17*-OE leaves and *MmNAC17*-RNAi leaves. The expression level of *MmNAC17* increased by about 6 times in *MmNAC17*-OE lines and decreased by 0.4 times in *MmNAC17*-RNAi compared to the empty control, respectively (Supplemental Fig. 15A). After being inoculated with *A. alternata* R1, *MmNAC17*-OE and *MmNAC17*-RNAi leaves displayed more milder and severe leaf disease symptoms compared to the empty control (Fig. 6C). Consistently, compared to the empty control, the lesion area was smaller and the cell membrane permeability was lower in the *MmNAC17*-OE leaves, while *MmNAC17*-RNAi leaves exhibited opposite phenotypes (Fig. 5D and Supplemental Fig. 15B). Furthermore, *MmNAC17* also significantly affected the Ca^2+^ level in leaves after pathogen infection, possibly activating various Ca^2+^ channels and promoting Ca^2+^ influx. Compared with the empty control, NMT indicated that the *MmNAC17*-OE leaves had a higher intracellular Ca^2+^ fluxes, while the intracellular Ca^2+^ fluxes in the MmNAC17-RNAi leaves were significantly reduced (Fig. 5E). It can be inferred that *MmNAC17* is an important gene responds to sorbitol and contributes to enhanceing plant disease resistance.

### *MmNAC17* directly binds to the promoter of *MmF3H* and *MmLAR* to activate their expression and promote the accumulation of the second tier signaling substance catechin

The above results have confirmed that both catechin and transcription factor *MmNAC17* exhibit disease resistance functions, therefore the regulatory relationship between them is worth studying. We analyzed most promoters of genes related to catechin synthesis and found that these synthase genes contain binding elements of different transcription factors. It is worth noting that there are numerous and densely distributed NAC binding sites on their promoters (Supplemental Fig. 16A). To determine the potential interaction between MmNAC17 and the above promoters, we performed a yeast one-hybrid (Y1H) assay. The 2 kb fragments of promoters were cloned into a reporter vector to be used as the bait, and the *MmNAC17-pGADT7* vector was the prey. Yeast cells co-transformed with the bait and prey grew on selective media lacking leucine with or without Aureobasidin A (AbA). The results showed that only yeast cells containing *proMmF3H* and *proMmLAR* could grow on SD/-Leu medium with 250 µg/ml AbA, indicating that MmNAC17 could bind to the promoter regions of *MmF3H* and *MmLAR* (Fig. 6A). In addition, we also examined whether MmbZIP1 and MmERF4 have an activating effect on these genes and found that neither of them can bind to their promoter regions (Supplemental Fig. 16B). This further confirms the important role of MmNAC17 in the regulation of catechin synthesis by sorbitol. To test the effect of MmNAC17 on the transcription of *MmF3H* and *MmLAR*, we used a transient expression assay in *M. micromalus* leaves. The *proMmF3H* or *proMmLAR* was fused to a GUS gene, and the expression of MmNAC17 was driven by the cauliflower mosaic virus (CaMV) 35 S promoter. When *proMmF3H*::GUS or *proMmLAR*::GUS was co-transformed with 35 S::MmNAC17, the GUS expression level was highly promoted when compared to that in *proMmF3H*::GUS or *proMmLAR*::GUS co-transformed with control vector (Fig. 6B). We also confirmed an activation of *proMmF3H* and *proMmLAR* by MmNAC17 using a luciferase-based reporter system in *Nicotiana benthamiana* leaves. The *proMmF3H* and *proMmLAR* were fused with a LUC gene, and MmNAC17 was constructed in the pGreenII-62SK vector, with its expression driven by the *CaMV 35 S* promoter. The results indicated that *MmNAC17* could enhance the expression of *proMmF3H: LUC* and *proMmLAR: LUC* in tobacco epidermal cells (Fig. 6C). NAC protein mainly regulates gene expression by binding to the NACRs core motifs (CACG and CGT (A/G)) in downstream genes (Yuan et al. 2019). Electrophoretic Mobility Shift Assay (EMSA) showed that purified NAC17 protein could bind to NACRs (CACGTG) on*MmF3H* promoter and NACRs (CACGTA) on *MmLAR* promoter, respectively, and the binding was disrupted by the addition of unlabeled competitive probes. Besides, the overexpression of *MmNAC17* in leaves also enhanced the transcription levels of *MmF3H* and *MmLAR* and promoted the accumulation of catechin (Fig. 6E-F). Overall, these results support the conclusion that MmNAC17 directly binds to the promoter regions of *MmF3H* and *MmLAR*. *MmF3H* and *MmLAR* are located upstream and downstream of the flavonoid biosynthesis pathway, respectively, the latter is at an important branch point that directly converts the substrate leucocyanidin to catechin (Fig. 3B). To identify the role of *MmF3H* and *MmLAR*, we suppressed their expression (Fig. 6G). As expected, low expression of *MmF3H* and *MmLAR* significantly impaired the biosynthesis of catechin in *M. micromalus* leaves (Fig. 6H). These results fully demonstrate that the promoting effect of MmNAC17 on catechin mainly depends on the expression of *MmF3H* and *MmLAR*.

### LncRNA809 acts as an upstream regulatory factor of MmNAC17

**Fig. 7:**
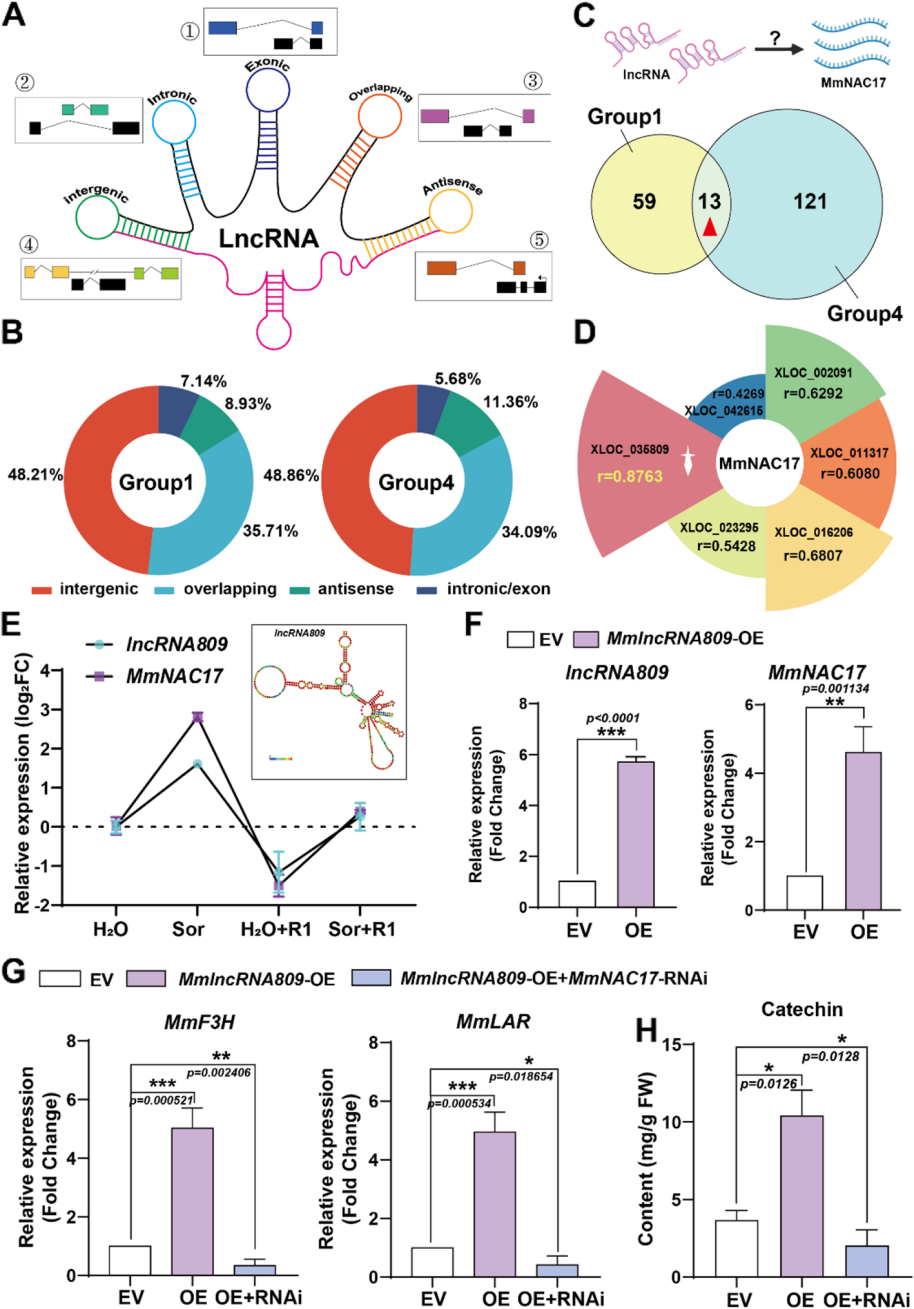
Long non-coding RNA lncRNA809 promotes the expression of MmNAC17 and promotes catechin biosynthesis. **A**-**D** LncRNA omics analysis and determination of lncRNA809. **A** Classification of lncRNAs. LncRNAs are divided into 5 categories according to the position of lncRNA and mRNA. **B** Proportional of 5 types of lncRNAs in Group 1 and Group 4. **C** Venn diagram of differentially expressed lncRNAs whose levels were significantly affected by sorbitol (H_2_O versus sorbitol and H_2_O + R1-infected leaves versus sorbitol + R1-infected leaves). **D** Analysis of collinearity between lncRNAs and MmNAC17. The lncRNA labeled with a red background has the highest correlation with MmNAC17. **E**-**G** Gene expression in different backgrounds. **E** Relative expression levels of lncRNA809 and MmNAC17 under sorbitol feeding and pathogen R1 infection. The structure of lncRNA809 is shown in the upper right corner. (F) Relative expression levels of lncRNA809 and MmNAC17 in leaves overexpressing lncRNA809. **G** Relative expression levels of *MmF3H* and *MmLAR* in lncRNA809-OE and lncRNA809-OE/MmNAC17-RNAi leaves. Transcripts were quantified by RT-qPCR. **H** The content of catechin in MmNAC17-OE and lncRNA809-OE/MmNAC17-RNAi leaves. Catechin were quantified by HPLC. The data is the mean ± SD (*n* = 3). *P < 0.05, **p < 0.01, ***p < 0.001, two-sided Student’s t-test

**Fig. 8:**
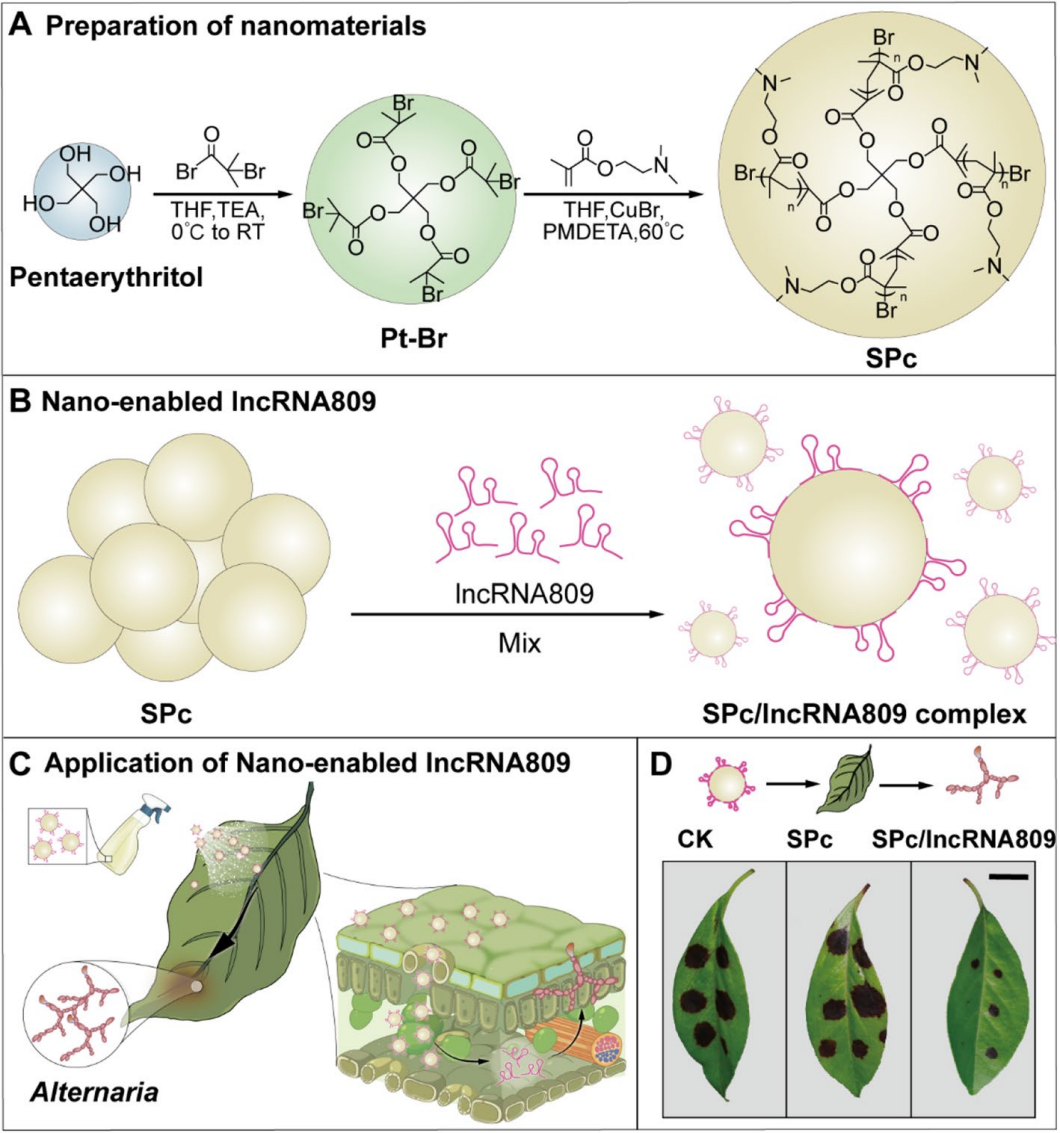
Preparation and characterization of SPc nanoparticles and SPc/lncRNA809. **A** The preparation process of SPc nanoparticles. **B** SPc nanocarriers combine with lncRNA809 through co-incubation to form SPc/lncRNA809 complexes. **C** Schematic illustration of the application of SPc/lncRNA809. Spray the prepared SPc/lncRNA809 solution onto the leaves of *M. micromalus*, and SPc/lncRNA809 is permeated into the leaves, followed by the release of lncRNA809 to inhibit pathogen invasion. **D** SPc/lncRNA809 complexes significantly enhanced the resistance of *M. micromalus* to *Alternaria* fungi. Scale bar, 1 cm

With the in-depth study of plant defense mechanisms, our understanding of transcriptional reprogramming during pathogen invasion, signal perception, and activation of defense genes has been expanded. Transcriptomic studies have shown that plants can transcribe a large number of long non-coding RNAs (lncRNAs), which are related to almost every biological processes in the organism. It can be said that diverse lncRNAs play important roles in plant development and stress response. Therefore, revealing the role of lncRNAs in sorbitol signaling and defense processes has become our research focus. Based on the omics data of *M. micromalus* leaves fed with sorbitol, we revealed that sorbitol regulated the expression of numerous lncRNAs, which raised the possibility that they are linked to catechin biosynthesis. Therefore, it is important to identify the lncRNAs that play a role in the synthesis of catechin and the defense response to *A. alternata* R1 in *M. micromalus*. According to the genomic location, the lncRNAs of omics data were categorized into five types: intronic lncRNAs, exonic lncRNAs, intergenic lncRNAs, antisense lncRNAs, and overlapping lncRNAs (Fig. 7A). Based on our lncRNA database, intergenic lncRNAs and overlapping lncRNAs constitute a larger proportion in Groups 1 and 4, approximately 48% and 35%, respectively (Fig. 7B). To further investigate the lncRNA that regulates the *MmNAC17* expression we mainly analyzed consistently expressed lncRNAs between Group 1 and Group 4. Thirteen lncRNAs existed in both groups (Fig. 7C) and six genes were significantly upregulated under both sorbitol feeding and pathogen inoculation. Notably, XLOC_035809 (named as *lncRNA809*) was predicted to have the highest correlation with *MmNAC17* (*r* = 0.8763) (Fig. 7D and Supplemental Fig. 18), therefore we supposed that *lncRNA809* might have a regulatory relationship with *MmNAC17*. To verify this, we identified the response ability of lncRNA809 to sorbitol and pathogens, and we found that the expression of lncRNA809 was significantly upregulated with sorbitol feeding, which was consistent with the expression of *MmNAC17* (Fig. 7E). Then, we cloned *lncRNA809* into the overexpression vector pROK2 and transiently transformed it into *M. micromalus* leaves to generate *lncRNA809*-OE transgenic leaves. As the expression level of *lncRNA809* significantly increased, the expression levels of *MmNAC17*, *MmF3H*, and *MmLAR* were also significantly induced (Fig. 7F-G), ultimately leading to a significant accumulation of catechin (Fig. 8H). The results indicated that *lncRNA809* can mediate the biosynthesis of the second-tier signaling substance, catechin. To confirm whether lncRNA809 regulates catechin biosynthesis by modulating the expression level of *MmNAC17*, we detected the expression levels of *MmF3H* and *MmLAR*, as well as the content of catechin, in the leaves co-transformed with *lncRNA809-OE* and *MmNAC17-RNAi.* The reduced expression of MmNAC17 led to a decrease in the expression levels of *MmF3H* and *MmLAR*, resulting in a significant reduction in catechin content. If MmNAC17 was inhibited, even when lncRNA809 was overexpressed at that time, it did not stimulate the synthesis of catechin (Fig. 7G-H). These findings indicated that *lncRNA809* must act through *MmNAC17* to respond to and transmit signals of sorbitol, and *MmNAC17* is a key downstream gene for lncRNA809 to regulate catechin biosynthesis.

In order to efficiently deliver lncRNA into plant cell, SPc was selected as the nanocarrier. SPc has been reported to have high intracellular delivery efficiency, as well as good biocompatibility and biodegradability. The SPc nanocarrier consists of a hydrophobic core and a hydrophilic shell, featuring positively charged tertiary amines in its side chains that enable binding to negatively charged nucleic acids (Fig. 8A). We co-incubated SPc nanocarriers with non-coding RNA, *lncRNA809*, for 30 min to promote their binding ability (Fig. 8B). Here, we developed a straightforward working model of the SPc/lncRNA809 complex: the prepared complex was sprayed onto the leaves, where it penetrated into the mesophyll cells through the stomata, followed by the release of lncRNA809 to inhibit pathogen invasion (Fig. 8C). To verify the application effect of the SPc/lncRNA809 complex, we inoculated the main pathogenic fungus onto the *Malus micromalus* leaves. It can be seen that SPc anlone did not enhance the resistance of the leaves, wherase the SPc/lncRNA809 complex significantly contributed to the resistant response (Fig. 8D). The SPc nanocarrier successfully delivered lncRNA809, thereby providing essential technical support for the subsequent work of this study.

### The primary signal, sorbitol regulates the secondary signal, catechin, through the lncRNA809-MmNAC17 module, thereby enhancing the disease resistance of *M. micromalus*

**Fig. 9:**
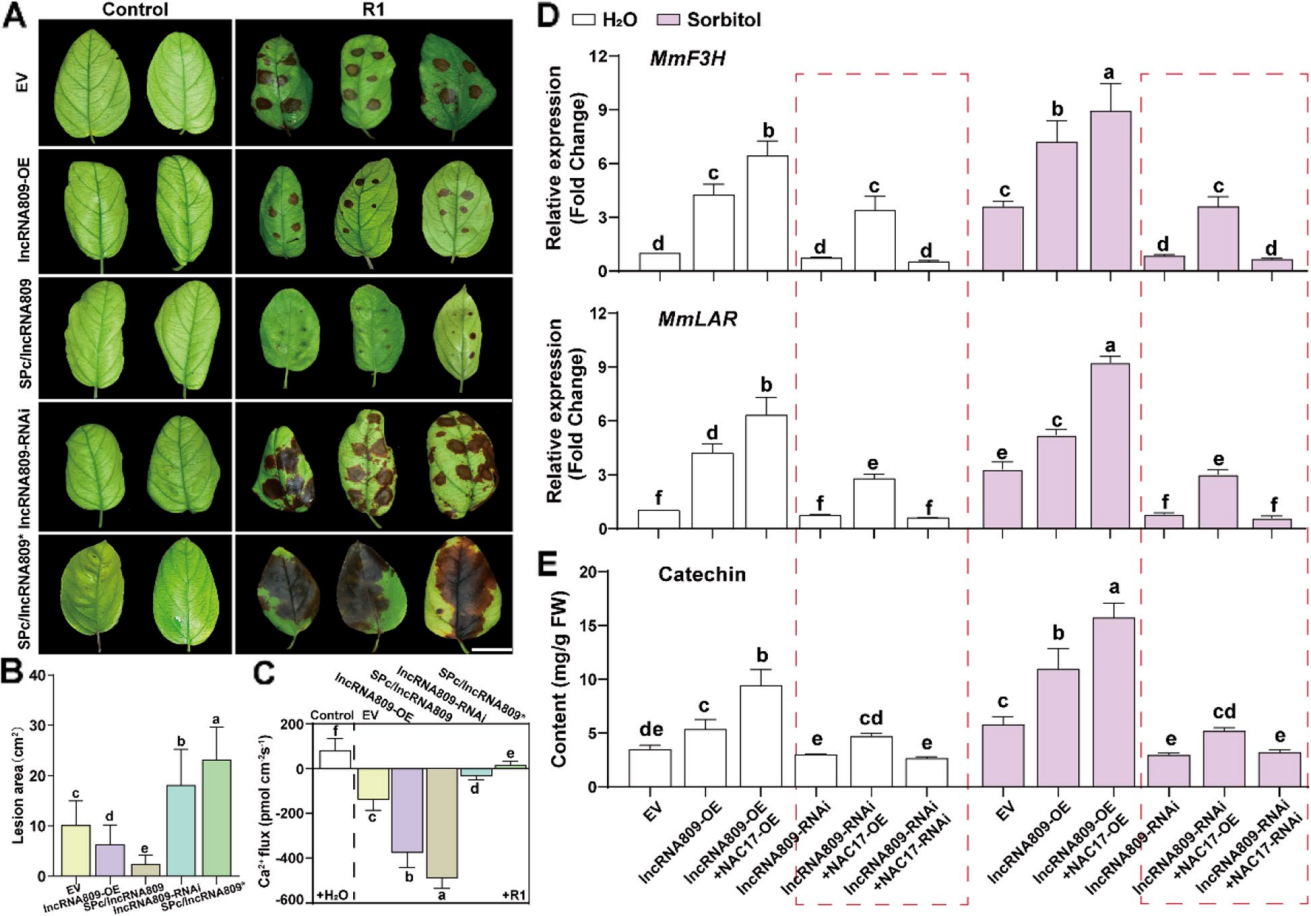
The key role of lncRNA809 in regulating catechin metabolism and enhancing *M. micromalus *disease resistance by sorbitol. **A**-**C** LncRNA809 regulates the disease resistance of leaves. Transgenic *M. micromalus* leaves were inoculated with R1 for 5 days. The representative phenotypes (**A**), quantification data of pathogen invasion severity (**B**) and Ca^2+^ flux (**C**) are shown. **D**-**E** Gene expression (**D**) and catechin content (**E**) under different transgenic leaf backgrounds. Young leaves that have completed gene transformation are cultured in a high humidity environment for 2 or 3 days. Then they were collected for RT-qPCR and HPLC analysis. ANOVA, Tukey’s multiple comparisons, *P *< 0.05, different letters indicate significant differences. Scale bar, 2 cm

The above results confirmed that *lncRNA809* is involved in the sorbitol signaling regulatory network to enchance the biosynthesis of catechin. To address the question of whether *lncRNA809* conferred disease resistance and acted as a pivotal upstream regulatory gene in the process of sorbitol regulating catechin synthesis we constructed *lncRNA809*-OE and *lncRNA809*-RNAi vectors respectively and transformed them into leaves for disease tolerance determination. Similarly, we first monitored the expression of *lncRNA809* in transgenic plant leaves before inoculating with R1. Compared with the empty control, the expression level of *lncRNA809* increased by about 7.5 times in *lncRNA809*-OE leaves and decreased by 0.3 times in *lncRNA809*-RNAi leaves (Supplemental Fig. 15C). After inoculation with R1, *lncRNA809*-RNAi leaves and *lncRNA809*-OE leaves displayed more severe and milder leaf disease symptoms, respectively, compared with the empty controls (Fig. 9A). Consistently, the lesion area was larger and the cell membrane permeability was higher in the *lncRNA809*-RNAi leaves, in contrast, the lesion area was smaller and the cell membrane permeability was lower in the *lncRNA809*-OE leaves, compared to the empty controls (Fig. 9B and Supplemental Fig. 15D). In addition, we detected the Ca^2+^ level in *lncRNA809*-OE and *lncRNA809*-RNAi leaves with or without pathogen infection using NMT analysis. Compared to the empty control, *lncRNA809*-OE leaves maintained a higher intracellular Ca^2+^ fluxes with R1 infection, while the intracellular Ca^2+^ fluxes in *lncRNA809*-RNAi leaves significantly decreased (Fig. 9C). Our data showed that *lncRNA809* promotes *M. micromalus* resistance to *A. alternata* R1 infection.

To determine whether *lncRNA809* plays an indispensable role in sorbitol signal transduction, we planned to inhibit the expression of *lncRNA809* and observe whether the disease resistance and the biosynthesis of catechin are still induced by sorbitol. Therefore, we set up different transformation combinations *lncRNA809*-OE group, *lncRNA809*-OE and *MmNAC17*-OE co-transformation group, *lncRNA809*-RNAi group, *lncRNA809*-RNAi and *MmNAC17*-OE co-transformation group, *lncRNA809*-RNAi and *MmNAC17*-RNAi co-transformation group. We fed different combinations of instant transgenic leaves with water and sorbitol, respectively, and completed the inoculation treatment at the same time. The leaves of the *lncRNA809*-OE group, *lncRNA809*-OE and *MmNAC17*-OE co-transformation group actively responded to sorbitol treatment and maintained a smaller lesion area and lower cell membrane permeability after pathogen infection. However, the leaves from the *lncRNA809*-RNAi group, *lncRNA809*-RNAi and *MmNAC17*-OE co-transformation group, *lncRNA809*-RNAi and *MmNAC17*-RNAi co-transformation group were not sensitive to sorbitol and did not change their lesion size and cell membrane permeability (Supplemental Fig. 19A-B). Feeding with sorbitol could still enhance the intracellular Ca^2+^ fluxes of different transgenic combination leaves before infection with R1 as detected by NMT. After pathogen infection, the intracellular Ca^2+^ fluxes in transgenic leaves with low expression of *lncRNA809* remained unchanged, while the intracellular Ca^2+^ fluxes in transgenic leaves of other combinations increased due to the resistance conferred by sorbitol (Supplemental Fig. 19C). This observation suggests that the expression level of *lncRNA809* determines the strength of the sorbitol signal in the immune process.

**Fig. 10:**
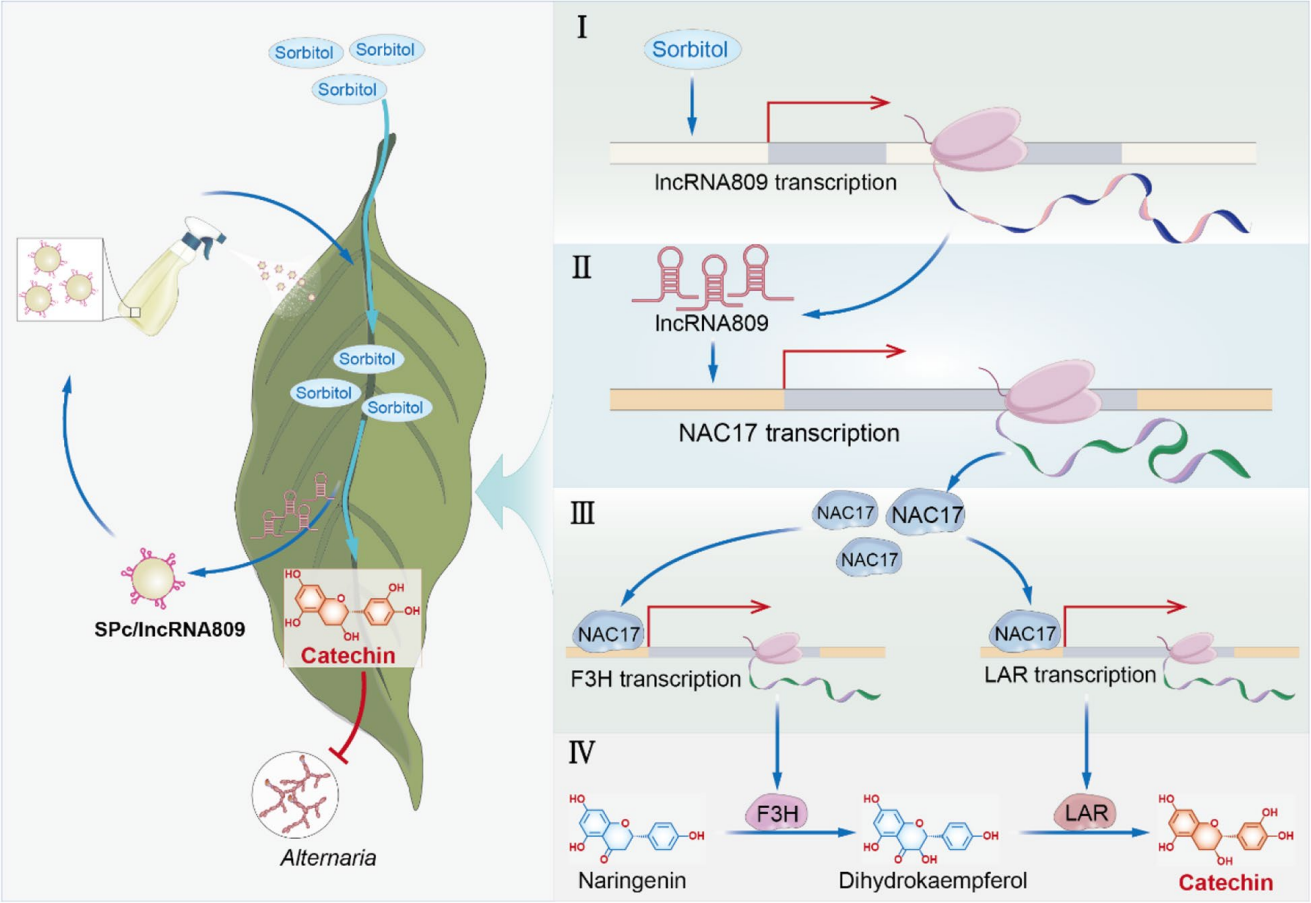
A proposed working model for regulating plant disease resistance by sorbitol-catechin cascade reaction. One-way solid arrows represent promotion. Solid terminated lines represent inhibition. Sorbitol enhances plants resistance to fungal infections by regulating the biosynthesis of catechin. The regulation of catechin biosynthesis by sorbitol is mainly achieved through the cascade reaction of lncRNA809-MmNAC17. The input of sorbitol enhances the transcription of lncRNA809, which can regulate the transcription factor MmNAC17 through *trans* action. MmNAC17 can bind to the promoters of F3H and LAR to activate their expression. The expression of F3H and LAR determines the level of catechin. When plants are exposed to pathogens, sorbitol transmits signals to catechin through a series of processes, ultimately activating plant immunity. At the same time, SPc nanomedicines derived from non-coding RNA lncRNA809 also have a good defense effect against *Alternaria* pathogens by mediating the biosynthesis of catechin

To further investigate whether the regulation of catechin biosynthesis by sorbitol also depends on *lncRNA809*, we also examined the expression levels of key flavonoid synthase genes and catechin accumulation in the leaves of different transgenic plants. The expression levels of *MmF3H* and *MmLAR* in the leaves of *lncRNA809*-OE group, *lncRNA809*-OE and *MmNAC17*-OE co-transformed group were significantly increased in response to the sorbitol signal. However, sorbitol did not alter the expression of *MmF3H* and *MmLAR* in *lncRNA809*-RNAi transgenic leaves (Fig. 9D). The content of catechin detected by HPLC showed the same trend (Fig. 9E). These data all supported the view that *lncRNA809* plays an indispensable role in the regulation of catechin metabolism pathway by sorbitol signaling. Therefore, we propose a viewpoint that sorbitol positively regulates the expression of catechin synthesis genes through the *lncRNA809-MmNAC17* module, thereby resisting against the *A. alternata* R1 fungal disease (Fig. 10). This expands our understanding of the role of sorbitol in the resistance of fruit trees to fungal diseases, especially as the upstream primary signal is transmitted through non-coding RNA to the secondary signal, the metabolite flavonoids, ultimately achieving the effect of disease resistance.

Based on the anti-disease function of lncRNA809, we further propose the efficient utilization of lncRNA through nanotechnology. We prepared SPc nanocarriers and combined them with lncRNA809 and lncRNA809* (dsRNA targeting lncRNA809 and inhibiting its expression) to form SPc/lncRNA809 (lncRNA809*) complexes. We first tested the effect of SPc/lncRNA809 (lncRNA809*) complex on the expression level of lncRNA809 in *M.micromalus*. In SPc/lncRNA809 leaves, the expression of lncRNA809 was significantly higher than in that of lncRNA809-OE leaves, while in SPc/lncRNA809* leaves, the expression of lncRNA809 was significantly lower than that of lncRNA809-RNAi leaves (Supplemental Fig. 15C). The application of the SPc nanocarriers has successfully achieved efficient delivery of lncRNA809. We further tested the application effect of SPc complex on the disease resistance of *M. micromalus*. The results showed that the SPc/lncRNA809 complex significantly enhanced the resistance of *M. micromalus* leaves to *A. alternata* R1 fungus, exhibiting a lighter leaf disease phenotype(Fig. 9A), smaller leaf damage area (Fig. 9B), lower cell membrane permeability (Supplemental Fig. 15D), and stronger calcium influx (Fig. 9C) than lncRNA809-OE leaves; The application of SPc/lncRNA809* complex showed the opposite effect, which was more severe than the performance of lncRNA809-RNAi leaves. In summary, nonbiological protectants enhance the plant’s defense response against pathogens, achieving sustainable green management of *A. alternata* R1 fungi from the perspective of pathogen inhibition and elevated plant defense.

## Discussion

As is well known, various factors can regulate plant defense, such as resistance gene-triggered immunity (Van Der Hoorn and Kourelis 2018), non-coding RNA(lncRNA, miRNA)-mediated immunity (Zhang et al. 2022; Cui et al. 2020), hormone-mediated immunity (Mishina and Zeier 2007; Tsuda et al. 2008), and secondary metabolite pathways (Ahuja et al. 2012; Bednarek 2012). In early research, we found that the primary metabolite sorbitol is not only a product of plant photosynthesis and a carbon source for pathogens, but also serves as a signal to regulate plant defense responses. Sorbitol regulated the expression of R gene*MdNLR16* to adjust apple resistance to *A. alternata*R1 fungi (Meng et al. 2018). This resistance induced by the photosynthetic product sorbitol provides new insights into plant defense mechanisms. Here, we regard the sorbitol as primary signal and observe that catechin biosynthesis genes*MmF3H* and *MmLAR* expression is enhanced by sorbitol, ultimately leading to increased resistance of *M. micromalus* to *A. alternata* R1. Hence, catechin as the main secondary metabolite flavonoids in response to diseases is identified as the secondary signal.

Long non-coding RNA *lncRNA809* and transcription factor *MmNAC17* are key genes involved in the regulation of catechin biosynthesis by sorbitol. MmNAC17 binds to the promoters of *MmF3H* and *MmLAR* in activating their expression in response to sorbitol. These findings reveal that the defense pathway mediated by sorbitol in the synthesis of secondary metabolites has become an important mechanism for plants to resist biological stress. The application of environmental-friendly fungicides, such as elicitors and botanical fungicides, to minimize the risk of exposure to the ecosystem has become a global trend. A star polymer (SPc) is constructed to deliver plant elicitors/botanical fungicides at a low production cost. The length of lncRNA exceeds 200 nucleotides, and their length and poor stability make them difficult to synthesize. Thus, we have constructed nanocarriers that encapsulate lncRNAs effectively to overcome the instability of lncRNAs, poor specificity, and their off-target effects.

How does sorbitol transmit signals when facing pathogen attacks? Based on our research, we believe that sorbitol, as an upstream signal, can control the output of multiple downstream signals, and propose a hierarchical regulatory network centered on sorbitol signals. The proposal of this viewpoint mainly stems from current research on the Gene Regulatory Network (GRN). In eukaryotes, environmental reactions and complex developmental processes are regulated by various hierarchical the GRNs (Song et al. 2016; Gerstein et al. 2012; Erwin and Douglas et al. 2009). The GRN model captures the intricate interplay of regulatory genes over time and space, thereby forming a broader hierarchical structure. In the GRN model, the structural genes associated with a metabolic pathway or biological process are used as inputs for the bottom layer, while all transcription factors (TFs) are employed as inputs for constructing the upper layers. This bottom-up algorithm has successfully obtained a hierarchical regulatory network centered on transcription factors (Wei 2019), which has been applied in plant growth, development, and stress response (Xu et al. 2022). By analogy, although no relevant mathematical model has yet been established centered on signaling substances, as the signaling roles of primary and secondary metabolites in plant resistance are gradually being explored, the speculation of cascade effects between primary and secondary metabolites will also become possible. In our study, sorbitol significantly increased the resistance of*M. micromalus* leaves to *A. alternata* R1. Under normal circumstances, R1 can rapidly expand on young leaves, while the expansion rate was inhibited after pretreatment with sorbitol on the leaves (Fig. 1A-B). In this process, we detected that a large number of genes exhibited significantly downregulated transcriptional levels, while many genes pre-treated with sorbitol had higher transcription levels than those treated with water (Fig. 2B). This indicates that sorbitol triggers transcriptional reprogramming of some genes under pathogen infection, keeping them at high expression, and these genes were significantly enriched in metabolic pathways such as amino sugar and nucleoside sugar metabolism, phenolpropanoid biosynthesis, starch and sucrose metabolism, plant hormone signal transduction, and flavonoid biosynthesis (Fig. 2D). Surprisingly, genes involved in flavonoid biosynthesis exhibited specificity, and they all exhibit upregulated responses induced by sorbitol (Fig. 2D and Supplemental Fig. 4A). Therefore, we believe that sorbitol may act as an upper-level signal to regulate the metabolism of sublayer signal flavonoids and plant disease resistance in plant immune signal transduction.

Early research on flavonoids mainly focused on leguminous plants, as they are known to be a rich source of flavonoids. In leguminous plants, flavonoids are a key signal for nodule formation in nitrogen fixation symbiosis, acting as inducers of nodulation and nodulation related genes in rhizobia (Cooper 2007). Chalcones, flavanones, isoflavones and flavones have been shown to exhibit*nod*gene induction activity in different leguminous-rhizobia interactions (Aoki 2000). Meanwhile, flavonoids are also considered effective substances in plant defense. Relevant evidences suggest that isoflavones exhibit broad-spectrum in vitro antibacterial activity (Dixon et al. 2002)and accumulate in infected tissues under pathogen attack, thereby qualifying them as important plant antitoxins (Aoki 2000). In addition, flavonoids have also been endowed with other biological significance. For example, the accumulation of flavones and anthocyanins in chrysanthemum is inhibited by high temperature, seriously weakening its ability to cope with high temperature and impacting flower color formation, thereby diminishing the commercial value of chrysanthemum (Zhou et al. 2021). When plants are exposed to drought environments, genes related to the synthesis of anthocyanin, genistein, and apigenin are strongly induced to alleviate their stress (Meng et al. 2021; Tapia et al. 2022). The attack of*Magnaporthe oryzae* can significantly enhance the biosynthesis of the flavonoid sakuranetin in rice, and the application of sakuranetin to *osnomt* mutants (transgenic lines with a low sakuranetin level) can rescue their resistance to *M. oryzae *(He et al. 2024). These findings indicate that the regulation of flavonoids by external pressure conditions is complex and species-specific. In this study, we investigated the regulatory effect of sorbitol on the flavonoid pathway in*M. micromalus* leaves and identified that catechin was significantly upregulated by sorbitol treatment or fungal attack (Fig. 3B-D). Catechin has been identified to have direct antifungal activity against fungal diseases. Catechin was first reported to act as an allelopathic substance in leguminous plants. It can trigger the production of reactive oxygen species in susceptible plants, leading to cell death and preventing pathogenic bacteria from absorbing nutrients (Bais et al. 2003). During the outbreak of apple rust, catechin also increases during the stage of disease expansion (Lu et al. 2017). In vitro, catechin directly inhibited the mycelial growth and spore germination of R1 in a concentration dependent manner (Supplemental Fig. 7 and Fig.4A-E). In plants, catechin treatment reduced the proportion of diseased leaves, and alleviated the increase in cell membrane permeability and Ca^2+^ influx caused by pathogen attack (Fig. 4F-I). Our findings underscore the role of flavonoid metabolite catechin in the process of sorbitol-mediated antifungal resistance in *M. micromalus*leaves. Catechin may function as signaling molecules to regulate intracellular signal transduction. At present, the claim that flavonoids regulate intracellular signal transduction in plants as signaling substances is receiving widespread experimental support. In addition to the discovery in the interaction between leguminous plants and rhizobia, the flavonoid compound hyperoside has also been reported to promote pollen tube growth in okra flower development by regulating actin depolymerization factors (Dong et al. 2021). Although the above studies have confirmed the signal role of flavonoids, it is still unclear how they regulate the plant defense process and may become a target for future research.

As the most abundant class of ncRNAs, lncRNAs serve as key regulatory factors for gene expression in various biological processes, but the functional role of many members in plants remains a mystery. Fortunately, high-resolution analysis of plant transcriptomes has provided a more comprehensive view of lncRNAs in plants. Research has shown that lncRNAs can perform their functions in *cis* or *trans*, manipulating plant immune responses by positively or negatively regulating functional genes in plants. When lncRNA and its target genes are located on the same chromosome and are very close, lncRNA plays a role in a *cis*manner. Conversely, when a lncRNA influences genes on different chromosomes, it operates in a trans manner (Kornienko et al. 2013). A recent study has demonstrated that lncRNA SABC1 in*Arabidopsis*suppresses plant immunity by inhibiting the transcription of its overlapping gene NAC3 (Liu et al. 2022). In addition, the transcriptional regulation of the immune response gene PR1 by long non-coding RNA ELENA1 enhances plant resistance to*Pst*DC3000 (Seo et al. 2017). This demonstrates that transcriptional regulation mediated by lncRNAs is a critical component of the plant immune response. In this work, we identified the transcriptional regulatory effect of lncRNA809 on*MmNAC17*. *MmNAC17* was a downstream target gene of lncRNA809, and their expression profiles were highly consistent under sorbitol treatment and pathogen attack. Overexpression of lncRNA809 positively regulated the transcription level of *MmNAC17* (Fig. 7E-F). The elevated expression of *MmNAC17* significantly promoted the accumulation of catechin, as it could bind to the promoters of F3H and LAR and regulated their transcription (Fig. 6). It is still unclear how lncRNAs successfully regulate plant immune responses, but targeting transcription factors is an effective way for lncRNAs to exert their biological functions effectively (Gil and Ulitsky 2020). Therefore, we could observe the active role of lncRNA809 on regulating catechin biosynthesis (Fig.7G-H). When the transcription of lncRNA809 was inhibited, the sorbitol signal cannot be transmitted to the secondary signal catechin (Fig. 8D-E), resulting no significant enhancement of leaf resistance (Supplemental Fig. 19). Therefore, we believe that the lncRNA809-NAC17 module is a key point in the cascade reaction between sorbitol signal and catechin signal, providing a framework for revealing how sorbitol metabolism regulates flavonoid metabolism in *Rosaceae* plants under pathogen attack.

In this study, we revealed how sorbitol alters catechin metabolism levels through lncRNA to resist fungal infection. When plants are exposed to pathogens, *lncRNA809* is maintained at a high expression level in leaves with high levels of sorbitol, and positively promotes the expression of transcription factor *MmNAC17*. *MmNAC17* further activates the expression of two synthase genes *MmF3H* and *MmLAR* on the flavonoid metabolism pathway and effectively promote the accumulation of flavonoid catechins. Catechin can inhibit the mycelial growth and spore germination of *A. alternata* R1, so leaves with high levels of catechin are more resistant (Fig. 9). In summary, this study indicates that a hierarchical regulation can be formed between sorbitol and catechin to resist *A. alternata* R1 diseases, and lncRNA809 and MmNAC17 are important regulatory genes that connect the two signaling factors.

## Materials and methods

### Plant materials and pathogen inoculation

Fully expanded young leaves from *Malus micromalus* trees planted on the campus of Beijing Forestry University were collected for relevant experiments. A culture of *A. alternata* strain R1 was initiated by plating stock culture stored at − 80 °C on a Potato Dextrose Agar medium at 25 °C. After 3 days, the actively growing hyphae were transferred to a new medium and grow for 7 days at 25℃. The culture was then filtered through two layers of nylon cloth. The conidial concentration was determined using a hemocytometer and the filtrate was adjusted to 1 × 10^6^ conidia per milliliter by adding sterile water.

For R1 infection, *M. micromalus* leaves were infected by drop inoculation with 10 µl R1 conidiospore suspension per leaf. The drop-inoculated leaves were placed in high humidity to complete infection. Disease severity was assessed by measuring lesion size (ImageJ software) and cell membrane permeability. For each leaf, the lesion area needs to be counted. Then, the percentage of lesion area to each leaf area was calculated. Based on the above percentage data, disease severity was classified into four grades: healthy, mild symptoms, moderate symptoms and severe symptoms. 40–50 leaves were used for statistical analysis of disease severity.

### Sorbitol feeding experiment and sorbitol content assays

Fully expanded and healthy leaves outdoors were collected and cleaned on the surface before being placed in a plastic culture dish (150 mm) with a layer of moist filter paper laid. 50mM sorbitol solution and a control solution (water) were prepared, and the petiole base was trimmed with scissor. Fixing the leaves so that the petioles are completely immersed in both solutions, and let them stand for 6 h. The feeding process is completed in an ultra clean platform, accompanied by a certain intensity of wind to ensure the maximum absorption efficiency of sorbitol. The Sorbitol Content Determination Kit (Solarbio, China) was used to detect the sorbitol content of leaves. Sorbitol can form a blue complex with copper ions in alkaline solutions, with a maximum absorption peak at a wavelength of 655 nm.

### Ca^2+^ flux measurement

NMT was used to measure the Ca^2+^ flux of leaves infected with pathogens and the effect of sorbitol or catechin on Ca^2+^flux as previously described (Luo et al. 2023; Yu et al. 2023). In brief, the leaf samples were immobilized in the testing solution (0.1 mM CaCl_2_, pH 6.0) for 30 min to achieve Ca^2+^ flux stability. The electrode tip was brought close to the mesophyll cell mass and maintained there until the signal stabilized. The effect of sorbitol or catechin on Ca^2+^ flux was tested. sorbitol or catechin was added transiently, and NMT data were recorded. The steady-state fluxes were continuously recorded every 6 s for 5 min. Data were obtained from at least three leaves for each treatment.

### Construction of lncRNA-seq and RNA-seq libraries

The leaves that had been treated with sorbitol and infected with pathogens for 7 days were divided into four groups: Healthy leaves fed with water, healthy leaves fed with sorbitol, leaves infected with R1 after sorbitol feeding and leaves infected with R1 after water feeding. Three biological replicates were designed for each group, with a total of 12 samples for whole-genome RNA-seq. RNA-seq was performed by Yung Biotechnology Co., Ltd.(Beijing, China). Total RNA was isolated from each leaves sample using TRIzol^®^ Reagent Plant Kits (Thermo, USA). Ribosomal RNA was removed using the Ribo-Zero Magnetic kit (Epicentre, ShangHai). Sequencing libraries were constructed using the TruseqTM RNA sample prep Kit for Illumina, according to the manufacturer’s instructions. The libraries were sequenced on the Illumina Novaseq 6000 platform.

### Identification of lncRNAs and prediction of its regulatory genes

The raw data were processed by filtering adaptors, removing the low-quality reads, and trimming the reads whose number of N bases accounted for more than 10% of the total (quality score, Q ≥ 30). The reference *Malus domestica* genome and the annotation files were downloaded from the NCBI database (ftp://ftp.ncbi.nlm.nih.gov/genomes/all/GCA/004/115/385/GCA_004115385.1_ASM411538v1). The genome index was built with Bowtie2 and clean reads were mapped to the *Malus domestica* genome using HISAT2. If the protein coding potential of a transcript is < 0, it means that the transcript cannot encode a protein and is considered non-coding. The lncRNAs *cis*-regulated target genes were predicted by Tbtools. Using genome annotation and genome browser to identify genes within a 10 kb upstream and downstream range of lncRNAs. The lncRNAs *trans*-regulated target genes were predicted using a Pearson correlation coefficient analysis between the expression of lncRNA and its protein coding genes. A Venn diagram was used to screen for the specific lncRNAs and mRNAs that had a significant difference in expression levels between the samples fed with sorbitol and samples fed with water while simultaneously having significant difference in expression levels between the infected samples after sorbitol feeding and those infected after water feeding. Using the Venn plot to screen for specific mRNA and lncRNAs with significant differences in expression levels between sorbitol fed and water fed samples, as well as significant differences in expression levels between samples infected after sorbitol feeding and those infected after water feeding. The screened genes were considered to have R1 resistance and respond to sorbitol.

### Identification of differentially expressed genes(DEGs)

The expression levels of lncRNA and the mRNA transcripts were estimated using StringTie and all mapped reads by StringTie. All RNA-seq datasets were respectively aligned to *Malus domestica* genome using HISAT2. The transcriptome from each dataset was then independently assembled using StringTie. The differential gene expression analyses were conducted using DESeq, based on a negative binomial distribution. Genes with Q ≤ 0.05 and |log2_ratio| ≥ 0.5 were considered to be differentially expressed genes.

### RT-qPCR analysis

Total RNA was extracted by using Eastep^®^ Super Total RNA Extraction Kit (Promega, USA) according to the manufacturer’s instructions. Agarose gel electrophoresis was used to detect the degradation and integrity of RNA, and NanoDrop 8000 (Thermo, USA) was used to detect the quality of RNA. cDNA was synthesized with a First-strand cDNA Synthesis Mix (LABLEAD, China) for qPCR. Quantitative PCR was performed by using a SYBR Premix Ex Taq Kit (TaKaRa) on a QuantStudio 6 Real-Time PCR system following the manufacturer’s instructions. Expression levels were normalized to the expression of *MmPR5*, which is stably expressed reference genes in *M. micromalus*. The relative fold change of genes was calculated using the 2^−△△CT^ method. The quantitative primers used in this study were generated by Sangon Biotech (https://www.sangon.com/primerDesign).

### Vector construction and instantaneous transformation of leaves

In order to overexpress MmNAC17 and lncRNA809 in plants, specific primers were used to amplify the ORF of MmNAC17 and the full-length sequence of lncRNA809, and they were cloned into the pROK2 vector driven by the 35 S promoter fusion with GFP-tag. In order to suppress the expression of MmNAC17 and lncRNA809 in plants, we amplified 80–200 bp specific fragments of MmNAC17 and lncRNA809 and cloned them into the pFGC5941 vector. For GUS expression system, the promoters of F3H and LAR were inserted into the pROK2 vector fused with GUS-tag to construct proMmF3H::GUS, proMmLAR::GUS reporter vectors, respectively. The construction of inhibitory expression vectors for F3H and LAR is consistent with the described methods for NAC17 and lncRNA809 mentioned above. The above constructed vectors were respectively introduced into the *Agrobacterium tumefaciens* strain GV3101. The *Agrobacterium tumefaciens* was shaken and cultured in YRK medium (YEP + 25 mg L^−1^ Rif + 50 mg L^−1^ kana) for 12 h. Subsequently, we collected the plaque and resuspend it with MS until the OD value is 0.4. 20 mM AS was added to activate the activity of Agrobacterium and allowed to stand for 2–3 h. If two genes are co-transformed into plant leaves, it is necessary to pre-mix the MS solution in a 1:1 ratio. The leaves were placed in MS solution containing the target gene and subjected to vacuum filtration for 20 min. After translation, the leaves were placed in a culture dish with high humidity and cultured for 2 days under 16 h of light/8 hours of darkness. The translation positivity rate was identified through RT-qPCR. Primers used are provided in Supplemental Table S1.

### Synthesis of SPc, dsRNA and lncRNA in vitro

SPc was synthesized according to the method described by Li et al. (2019). The star initiator Pt-Br was synthesized using pentaerythritol as raw material. the polymerization of star initiator with DMAEMA (2-(Dimethylamino) ethyl methacrylate) and dried tetrahydrofuran was carried out under nitrogen atmosphere. After removal of THF in a rotary evaporator, the water was added to dialyze and purify the crude polymer, which was repeated 4 times to obtain the final SPc product.

We have previously cloned the 80–200 bp specific fragment of lncRNA809 into the MCS1 and MCS2 regions of the pFGC5941 vector, respectively. The above plasmids were used as templates, and primers were designed for gene fragment amplification at the upstream position of MCS1 and downstream position of MCS2, respectively. The amplification product is named lncRNA809*. The amplified lncRNA809* fragment was cloned into pMD19T-vector and transformed into *E. coli* DH5α. The plasmid (lncRNA809*-T) was extracted and verified by Sanger sequencing. LncRNA809* and lncRNA809 plasmids with T7 promoter were used as in *vitro* transcription templates, and then a large amount of RNA products were efficiently obtained through in vitro transcription reaction using T7 High Yield RNA Transcription Kit (Vazyme, China).

### Y1H assay

The leaf DNA extracted by CTAB method was used as a template to amplify 2000 bp of the CHS, F3H, F3’H, LAR, LDOX promoter region, which were cloned into the pAbAi vector. The ORFs of MmNAC17, MmbZIP1 and MmERF4 were inserted into the pGADT7-Rec2 (AD) vector. These constructs or corresponding empty vectors were co-transformed into the yeast strain Y1HGold, which was subsequently incubated at 29 °C on the synthetic dropout (SD) medium lacking Leu. It was spotted on selective media with 250 ng mL^−1^ AbA following the manufacturer’s instructions. Primers used are provided in Supplemental Table S1.

### Dual-LUC assay

The promoter regions of *MmF3H* and *MmLAR* were inserted as reporter genes into the pGreenII 0800-LUC vector. The coding sequence of MmNAC17 was cloned into the pGreenII-62-SK vector. The effector gene and reporter gene were transformed into *Agrobacterium tumefaciens strain* GV3101 and co-infiltrated into *N. benthamiana* leaves. After 48 h, the luciferin substrate was sprayed onto the surface of leaves and its luminescence was detected with an imaging system (Tanon, Shanghai). The primers used are listed in Supplemental Table S1.

### Protein purification and EMSA experiment

The CDS sequence of NAC17 was constructed into the pET-30a vector, and the recombinant plasmid was subsequently transformed into *Escherichia coli* BL21(DE3). When the bacterial solution was cultured at 37℃ until the OD reached 0.8, the protein was induced to overexpress by adding 0.5mM Isopropyl-beta-D-thiogalactopyranoside (IPTG). After 16 h, the bacteria were collected and resuspended. The components of the resuspension buffer are 1 M Tris HCl (pH 7.5), 0.5 M NaCl, 20mM imidazole, and 100 mM Phenylethenesulfonyl fluoride (PMSF). The supernatant was collected by centrifugation at 12,000 rpm for 30 min after sonication. The proteins in the supernatant were collected by Ni-NTA agarose. The EMSA experiment was conducted using a chemiluminescence EMSA kit (Beyotime Biotechnology, China). Biotin-labeled DNA fragments containing the NACR were used as probes, and the unlabeled DNA fragments were used as competitive probes. The mutation probe is a DNA sequence in which all NACR core motifs have been mutated to A.

### Determination of electrolyte leakage in leaves after pathogen inoculation

The leaves inoculated with pathogens and the control leaves were washed with clean water and cut into 1 cm×1 cm sizes. They were placed in 50 ml tubes filled with distilled water and stood at 37℃ for 24 h. The conductivity meter (Leci, DDS-307) was used to measure and record the value E1. Subsequently, the test tube was placed in a boiling water bath at 100℃ for 15 min. When that was completed, it was cooled to room temperature and the value E2 was recorded for the second measurement. Empty distilled water was used as a control, and the values measured before and after boiling were recorded respectively as E0 and EL0. The relative conductivity (REC) is calculated according to the following formula: REC (L)=(E1-E0)/(E2-EL0).

Detection of flavonoids by high performance liquid chromatography (HPLC) leaves inoculated with pathogens for 7 days or leaves transformed instantaneously for 3 days are collected. 2 g of leaves were weighed and ground, and then 20 ml 70% methanol solution was used to prepare flavonoid extract. The target metabolite was analyzed on an Agilent 1260 liquid chromatography system equipped with a Gemini C18 column ( 250 × 4.6 mm id, 5 μm, Phenomenex, USA). The detection wavelength is 254 nm. Chromatographic detection conditions: Mobile phase A is 0.1% formic acid, and mobile phase B is methanol solution. Gradient elution procedure: 0 ~ 20 min, 45 ~ 65% B; 20–25 min, 65 ~ 80% B; 25 ~ 60 min, 80 ~ 90% B. Gradient elution procedure: 0 ~ 10 min, 35 ~ 45% B; 10 ~ 20 min, 45 ~ 51% B; 20 ~ 35 min, 51 ~ 80% B; 35 ~ 36 min, 80 ~ 100%. The column temperature is 30℃. The injection volume of all samples was 20 ul, and the peak area was normalized to the control group for comparison.

### The inhibitory effect of flavonoids on the growth of pathogenic fungi R1

Different types of flavonoid standard solutions were prepared and added to potato solid culture medium according to concentration settings, with flavonoid free solid culture medium as the control. R1 was inoculated into the above-mentioned culture medium and cultured at 28℃, and the fungal growth diameter was recorded. R1 cultured for 96 h were selected to prepare spore suspensions, and the spore count and germ tube length were observed under an inverted microscope. ImageJ is used to statistically analyze the length of spore germ tubes.

### Statistical analysis

All data are shown as means ± standard deviation (SD) from at least three biological repeats or from three technical replicates in one of three experiments with similar results. Student’s T test was used for comparing means between two samples (*p < 0.05, **p < 0.01, ***p < 0.001, ****p < 0.0001). One-way analysis of variance (ANONA) was used for testing the significance of the difference among different group means (different lowercase letters indicate significant differences, *p* < 0.05).

## Supplementary Information

Supplementary Material 1. Supplemental table S1. The primers used in this study.

Supplementary Material 2. Water-fed healthy leaves.

Supplementary Material 3. Sorbitol-fed healthy leaves.

Supplementary Material 4. Water-fed and R1-infected leaves.

Supplementary Material 5. Sorbitol-fed and R1-infected leaves

Supplementary Material 6. The molecular mechanism of sorbitol enhancing disease resistance in *M. micromalus*.

Supplementary Material 7: Supplemental Figure 1. Isolation and identification of the main pathogenic fungus R1 from *M. micromalus*. (A) The isolation process of pathogenic fungi from *M. micromalus*. (B) Pathogenicity identification of R1, R2, and R3. Scale bar, 1 cm. (C) Phylogenetic tree analysis of R1. R1 belongs to the genus *A. alternata *R1. Supplemental Figure 2. The content of sorbitol in the leaves of *M. micromalus* fed with exogenous sorbitol is detected. The data is the mean ± SD (*n*=3). *p < 0.05, two-sided Student’s t-test. Supplemental Figure 3. The GO pathways that are enriched by differentially expressed genes regulated by the pathogen R1. Group 2, H_2_O vs H_2_O+R1. Group 3, sorbitol vs sorbitol+R1. Here are the top 15 entries with *p *< 0.05. Supplemental Figure 4. The KEGG pathways that are enriched by differentially expressed genes. (A) The KEGG pathways of downregulated genes in Group 1 and Group 4 are shown. (B) The KEGG pathways of upregulated and downregulated genes in Group 2 and Group 3 aredisplayed respectively. Supplemental Figure 5. A heatmap with the expression level of genes related to flavonoid synthesis.Supplemental Figure 6. Family member analysis of genes related to flavonoid synthesis. Supplemental Figure 7. The concentration of catechin that inhibit the growth of pathogens is determined.The antibacterial effect (A), pathogen growth diameter (B), and spore count (C) are shown. Scale bar, 2 cm. Supplemental Figure 8. The antibacterial effects of naringenin in vitro. R1 was inoculated onto PDA medium with naringenin and cultured at 25℃ for 96 hours. The growth process (A) and spore morphology (C) of pathogen R1 are displayed in the presence or absence of naringenin. Scale bars, 4.5 cm (A) and 20 μm (C). The growth diameter (B), spore quantity (D), and spore germination (E) are also statistically analyzed. Supplemental Figure 9. The antibacterial effects of quercetin in vitro. R1 was inoculated onto PDA medium with quercetin and cultured at 25℃ for 96 hours. The growth process (A) and spore morphology (C) of pathogen R1 are displayed in the presence or absence of quercetin. Scale bars, 4.5 cm (A) and 20 μm (C). The growth diameter (B), spore quantity (D), and spore germination (E) are also statistically analyzed. Supplemental Figure 10. The antibacterial effects of isorhamnetin in vitro. R1 was inoculated onto PDA medium with isorhamnetin and cultured at 25℃ for 96 hours. The growth process (A) and spore morphology (C) of pathogen R1 are displayed in the presence or absence of isorhamnetin. Scale bars, 4.5 cm (A) and 20 μm (C). The growth diameter (B), spore quantity (D), and spore germination (E) are also statistically analyzed. Supplemental Figure 11. The antibacterial effects of isoquercitrin in vitro. R1 was inoculated onto PDA medium with isoquercitrin and cultured at 25℃ for 96 hours. The growth process (A) and spore morphology (C) of pathogen R1 are displayed in the presence or absence of isoquercitrin. Scale bars, 4.5 cm (A) and 20 μm (C). The growth diameter (B), spore quantity (D), and spore germination (E) are also statistically analyzed. Supplemental Figure 12 The antibacterial effects of dihydroquercetin in vitro. R1 was inoculated onto PDA medium with dihydroquercetin and cultured at 25℃ for 96 hours. The growth process (A) and spore morphology (C) of pathogen R1 are displayed in the presence or absence of dihydroquercetin. Scale bars, 4.5 cm (A) and 20 μm (C). The growth diameter (B), spore quantity (D), and spore germination (E) are also statistically analyzed.Supplemental Figure 13. The antibacterial effects of epicatechin in vitro. R1 was inoculated onto PDA medium with epicatechin and cultured at 25℃ for 96 hours. The growth process (A) and spore morphology (C) of pathogen R1 are displayed in the presence or absence of epicatechin. Scale bars, 4.5 cm (A) and 20 μm (C). The growth diameter (B), spore quantity (D), and spore germination (E) are also statistically analyzed. Supplemental Figure 14. The expression levels of transcription factors are shown by heatmap. Supplemental Figure 15. Expression levels of MmNAC17 and lncRNA809 and cell membrane permeability of leaves under pathogen R1 infection. The relative expression levels of MmNAC17 and lncRNA809 (A, C). Transcripts are quantified by RT-qPCR. The data is the mean ± SD (*n*=3). **p < 0.01, ***p < 0.001, two-sided Student’s t-test. The cell membrane permeability of leaves (B, D). ANOVA, Tukey’s multiple comparisons, *P*<0.05, different letters indicate significant differences. Supplemental Figure 16. Prediction of promoter binding elements for flavonoid synthesis related genes and Y1H assays. (A) The transcription factor binding sites predicted using the plantpan3.0 database are displayed. (B) Y1H assay showing no interaction between transcription factors MmbZIP1 and MmERF4 and promoter fragments of flavonoid synthesis related genes. Empty pGADT7 vectors were used as a negative control. Supplemental Figure 17. The effect of overexpression of MmNAC17 on the biosynthesis of other flavonoids. Supplemental Figure 18. A heatmap of differentially expressed lncRNAs. Supplemental Figure 19. The lesion area, cell membrane permeability, and calcium ion flux of transgenic leaves under different backgrounds. The data is the mean ± SD (*n*=3). *p < 0.05, **p < 0.01,***p < 0.001, ****p < 0.0001, ns, not significant, two-sided Student’s t-test.

## Data Availability

The data that support the findings of this study are available from the corresponding author upon reasonable request.

## Abbreviations

*M. micromalus*: *Malus micromalus*
*A. alternata*: *Alternaria alternata*
*N. benthamiana*: *Nicotiana benthamiana*
NMT: Noninvasive Microtest Technology
DEGs: Differentially expressed genes
GO: Gene ontology
KEGG: Kyoto Encyclopedia of Genes and Genomes
EMSA: Electrophoretic Mobility Shift Assay
CHS: Chalcone Synthase
F3H: Flavonoid 3-hydroxylase
F3’H: Flavonoid 3’-hydroxylase
ANS: Anthocyanidin Synthase
LDOX: Leucoanthocyanidin O-methyltransferase
LAR: Leucoanthocyanidin Reductase
HPLC: High performance liquid chromatography
LncRNA809*: a dsRNA targeting lncRNA809

## References

[CR1] Ahuja I, Kissen R, Bones AM. Phytoalexins in defense against pathogens. Trends Plant Sci. 2012;17:73. 22209038 10.1016/j.tplants.2011.11.002

[CR2] Aoki T, Akashi T, Ayabe SI. Flavonoids of leguminous plants: structure, biological activity, and biosynthesis. J Plant Res. 2000;113:475.

[CR3] Bais HP, Vepachedu R, Gilroy S, Callaway RM, Vivanco JM. Allelopathy and exotic plant invasion: from molecules and genes to species interactions. Science. 2003;301:1377. 12958360 10.1126/science.1083245

[CR4] Bednarek P. Chemical warfare or modulators of defence responses–the function of secondary metabolites in plant immunity. Curr Opin Plant Biol. 2012;15:407. 22445190 10.1016/j.pbi.2012.03.002

[CR5] Bilir Ö, Telli O, Norman C, Budak H, Hong Y, Tör M. Small RNA inhibits infection by downy mildew pathogen *Hyaloperonospora Arabidopsidis*. Mol Plant Pathol. 2019;20:1523. 31557400 10.1111/mpp.12863 PMC6804343

[CR6] Bjarnsholt T, Ciofu O, Molin S, Givskov M, Høiby N. Applying insights from biofilm biology to drug development—can a new approach be developed? Nat Rev Drug Discovery. 2013;12:791. 24080700 10.1038/nrd4000

[CR7] Cooper JE. Early interactions between legumes and rhizobia: disclosing complexity in a molecular dialogue. J Appl Microbiol. 2007;103:1355. 17953546 10.1111/j.1365-2672.2007.03366.x

[CR8] Cui C, Wang JJ, Zhao JH, Fang YY, He XF, Guo HS, et al. A *Brassica* miRNA regulates plant growth and immunity through distinct modes of action. Mol Plant. 2020;13:231. 31794845 10.1016/j.molp.2019.11.010

[CR9] Dixon RA, Achnine L, Kota P, Liu CJ, Reddy MS, Wang L. The phenylpropanoid pathway and plant defence—a genomics perspective. Mol Plant Pathol. 2002;3:371. 20569344 10.1046/j.1364-3703.2002.00131.x

[CR10] Dong B, Yang Q, Song Z, Niu L, Cao H, Liu T, et al. Hyperoside promotes pollen tube growth by regulating the depolymerization effect of actin-depolymerizing factor 1 on microfilaments in okra. Hortic Res. 2021;8:145. 34193835 10.1038/s41438-021-00578-z PMC8245483

[CR11] Erwin DH, Davidson EH. The evolution of hierarchical gene regulatory networks. Nat Rev Genet. 2009;10:141. 19139764 10.1038/nrg2499

[CR12] Gerstein MB, Kundaje A, Hariharan M, Landt SG, Yan KK, Cheng C, et al. Architecture of the human regulatory network derived from ENCODE data. Nature. 2012;489:91. 22955619 10.1038/nature11245 PMC4154057

[CR13] Gil N, Ulitsky I. Regulation of gene expression by *cis*-acting long non-coding RNAs. Nat Rev Genet. 2020;21:102. 31729473 10.1038/s41576-019-0184-5

[CR14] Hao X, Wang S, Fu Y, Liu Y, Shen H, Jiang L, et al. The WRKY46–MYC2 module plays a critical role in e-2-hexenal-induced anti-herbivore responses by promoting flavonoid accumulation. Plant Commun. 2024;5:100734. 10.1016/j.xplc.2023.100734 PMC10873895 37859344

[CR15] He Y, Zhao Y, Hu J, Wang L, Li L, Zhang X, et al. The OsBZR1–OsSPX1/2 module fine-tunes the growth–immunity trade-off in adaptation to phosphate availability in rice. Mol Plant. 2024;17:258. 38069474 10.1016/j.molp.2023.12.003

[CR16] Hertel W, Peschel G, Ozegowski JH, Müller PJ. Inhibitory effects of triterpenes and flavonoids on the enzymatic activity of hyaluronic acid-splitting enzymes. Archiv Der Pharmazie: Int J Pharm Med Chem. 2006;339:313. 10.1002/ardp.200500216 16718670

[CR17] Kornienko AE, Guenzl PM, Barlow DP, Pauler FM. Gene regulation by the act of long non-coding RNA transcription. BMC Biol. 2013;11:59. 23721193 10.1186/1741-7007-11-59 PMC3668284

[CR18] Kourelis J, van der Hoorn RAL. Defended to the nines: 25 years of resistance gene cloning identifies nine mechanisms for R protein function. Plant Cell. 2018;30:285. 29382771 10.1105/tpc.17.00579 PMC5868693

[CR19] Kung JT, Colognori D, Lee JT. Long noncoding RNAs: past, present, and future. Genetics. 2013;193:651. 23463798 10.1534/genetics.112.146704 PMC3583990

[CR20] Lee YS, Maple R, Dürr J, Dawson A, Tamim S, Del Genio C, et al. A transposon surveillance mechanism that safeguards plant male fertility during stress. Nat Plants. 2021;7:34. 33398155 10.1038/s41477-020-00818-5

[CR21] Li J, Qian J, Xu Y, Yan S, Shen J, Yin M. A facile-synthesized star polycation constructed as a highly efficient gene vector in pest management. ACS Sustain Chem Eng. 2019;6:6316.

[CR22] Li C, Meng D, Piñeros MA, Mao Y, Dandekar AM, Cheng L. A sugar transporter takes up both hexose and sucrose for sorbitol-modulated in vitro pollen tube growth in apple. Plant Cell. 2020;32:449. 31826966 10.1105/tpc.19.00638 PMC7008483

[CR23] Liu N, Xu Y, Li Q, Cao Y, Yang D, Liu S, et al. A lncRNA fine-tunes salicylic acid biosynthesis to balance plant immunity and growth. Cell Host Microbe. 2022;30:1124. 35908550 10.1016/j.chom.2022.07.001

[CR24] Lu Y, Bu Y, Tian J, Yao Y. Flavonoid accumulation plays an important role in the rust resistance of Malus plant leaves. Front Plant Sci. 2017;8:1286. 28769974 10.3389/fpls.2017.01286 PMC5514348

[CR25] Luo X, Wang Z, Wang C, Yue L, Tao M, Elmer WH, et al. Nanomaterial size and surface modification mediate disease resistance activation in cucumber (*Cucumis sativus*). ACS Nano. 2023;17:4871. 36871293 10.1021/acsnano.2c11790

[CR26] Meng D, Li C, Park HJ, González J, Wang J, Dandekar AM, et al. Sorbitol modulates resistance to *Alternaria alternata* by regulating the expression of an NLR resistance gene in apple. Plant Cell. 2018;30:1562. 29871985 10.1105/tpc.18.00231 PMC6096587

[CR27] Meng D, He M, Bai Y, Xu H, Dandekar AM, Fei Z, et al. Decreased sorbitol synthesis leads to abnormal stamen development and reduced pollen tube growth via an MYB transcription factor, MdMYB39L, in apple (*Malus domestica*). New Phytol. 2018;217:641. 29027668 10.1111/nph.14824

[CR28] Meng D, Dong B, Niu L, Song Z, Wang L, Amin R, et al. The pigeon pea CcCIPK14-CcCBL1 pair positively modulates drought tolerance by enhancing flavonoid biosynthesis. Plant J. 2021;106:1278. 33733535 10.1111/tpj.15234

[CR29] Mishina TE, Zeier J. Pathogen-associated molecular pattern recognition rather than development of tissue necrosis contributes to bacterial induction of systemic acquired resistance in *Arabidopsis*. Plant J. 2007;50:500. 17419843 10.1111/j.1365-313X.2007.03067.x

[CR30] Palos K, Yu L, Railey CE, Nelson Dittrich AC, Nelson ADL. Linking discoveries, mechanisms, and technologies to develop a clearer perspective on plant long noncoding RNAs. Plant Cell. 2023;35:1762. 36738093 10.1093/plcell/koad027 PMC10226578

[CR31] Qi G, Chen H, Wang D, Zheng H, Tang X, Guo Z, et al. The BZR1-EDS1 module regulates plant growth-defense coordination. Mol Plant. 2021;14:2072. 34416351 10.1016/j.molp.2021.08.011

[CR32] Rabiey M, Welch T, Sanchez-Lucas R, Stevens K, Raw M, Kettles GJ, et al. Scaling-up to understand tree–pathogen interactions: a steep, tough climb or a walk in the park? Curr Opin Plant Biol. 2022;68:102229. 10.1016/j.pbi.2022.102229 35567925

[CR33] Rütschlin S, Böttcher T. Inhibitors of bacterial swarming behavior. Chemistry–A Eur J. 2020;26:964. 10.1002/chem.201901961 PMC7027876 31268192

[CR34] Seo JS, Sun HX, Park BS, Huang CH, Yeh SD, Jung C, et al. ELF18-induced long-noncoding RNA associates with mediator to enhance expression of innate immune response genes in *Arabidopsis.* Plant Cell. 2017;29:1024. 10.1105/tpc.16.00886 PMC5466027 28400491

[CR35] Song L, Huang SC, Wise A, Castanon R, Nery JR, Chen H, et al. A transcription factor hierarchy defines an environmental stress response network. Science. 2016;354:598. 10.1126/science.aag1550 PMC5217750 27811239

[CR36] St Laurent G, Wahlestedt C, Kapranov P. The Landscape of long noncoding RNA classification. Trends Genet. 2015;31:239. 25869999 10.1016/j.tig.2015.03.007 PMC4417002

[CR37] Tan X, Li S, Hu L, Zhang C. Genome-wide analysis of long non-coding RNAs (lncRNAs) in two contrasting rapeseed (*Brassica napus* L.) genotypes subjected to drought stress and re-watering. BMC Plant Biol. 2020;20:81. 10.1186/s12870-020-2286-9 PMC7032001 32075594

[CR38] Tapia G, Castro M, Gaete-Eastman C, Figueroa CR. Regulation of anthocyanin biosynthesis by drought and UV-B radiation in wild tomato (*Solanum peruvianum*) fruit. Antioxidants. 2022;11:1639. 36139713 10.3390/antiox11091639 PMC9495367

[CR39] Tsuda K, Sato M, Glazebrook J, Cohen JD, Katagiri F. Interplay between MAMP-triggered and SA-mediated defense responses. Plant J. 2008;53:763. 18005228 10.1111/j.1365-313X.2007.03369.x

[CR40] Wang MY, Chen JB, Wu R, Guo HL, Chen Y, Li ZJ, et al. The plant immune receptor SNC1 monitors helper NLRs targeted by a bacterial effector. Cell Host Microbe. 2023;31:1792. 37944492 10.1016/j.chom.2023.10.006

[CR41] Wei H. Construction of a hierarchical gene regulatory network centered around a transcription factor. Brief Bioinform. 2019;20:1021. 29186304 10.1093/bib/bbx152

[CR42] Xu H, Liu P, Wang C, Wu S, Dong C, Lin Q, et al. Transcriptional networks regulating suberin and lignin in endodermis link development and ABA response. Plant Physiol. 2022;190:1165. 35781829 10.1093/plphys/kiac298 PMC9516719

[CR43] Xu HX, Meng D, Yang Q, Chen T, Qi M, Li XY, et al. Sorbitol induces flower bud formation via the MADS-box transcription factor EjCAL in loquat. J Integr Plant Biol. 2023;65:1241. 36541724 10.1111/jipb.13439

[CR44] Yang T, Ma H, Zhang J, Wu T, Song T, Tian J, et al. Systematic identification of long noncoding RNA’s expressed during light-induced anthocyanin accumulation in apple fruit. Plant J. 2019;100:572. 31344284 10.1111/tpj.14470

[CR45] Yang Q, Dong B, Wang L, Song Z, Niu L, Li H, et al. CDPK6 phosphorylates and stabilizes MYB30 to promote hyperoside biosynthesis that prolongs the duration of full-blooming in okra. J Exp Bot. 2020;71:4042. 32249299 10.1093/jxb/eraa174

[CR46] Yang X, Li Y, Yu R, Zhang L, Yang Y, Xiao D, et al. Integrated transcriptomic and metabolomic profiles reveal adaptive responses of three poplar varieties against the bacterial pathogen* Lonsdalea Populi*. Plant Cell Environ. 2023;46:306. 36217265 10.1111/pce.14460

[CR47] Yu X, Xie Y, Luo D, Liu H, de Oliveira MVV, Qi P, et al. A phospho-switch constrains BTL2-mediated phytocytokine signaling in plant immunity. Cell. 2023;186:2329. 37192618 10.1016/j.cell.2023.04.027 PMC10281528

[CR48] Yuan X, Wang H, Cai J, Bi Y, Li D, Song F. Rice NAC transcription factor ONAC066 functions as a positive regulator of drought and oxidative stress response. BMC Plant Biol. 2019;19:278. 31238869 10.1186/s12870-019-1883-y PMC6593515

[CR49] Zhang D, Gao Z, Zhang H, Yang Y, Yang X, Zhao X, et al. The MAPK-Alfin-like 7 module negatively regulates ROS scavenging genes to promote NLR-mediated immunity. Proc Natl Acad Sci. 2023;120:e2214750120. 10.1073/pnas.2214750120 PMC9934166 36623197

[CR50] Zhang G, Chen D, Zhang T, Duan A, Zhang J, He C. Transcriptomic and functional analyses unveil the role of long non-coding RNAs in anthocyanin biosynthesis during sea buckthorn fruit ripening. DNA Res. 2018;25:465. 29873696 10.1093/dnares/dsy017 PMC6191307

[CR51] Zhang L, Wang M, Li N, Wang H, Qiu P, Pei L, et al. Long noncoding RNAs involve in resistance to *Verticillium Dahliae*, a fungal disease in cotton. Plant Biotechnol J. 2018;16:1172. 29149461 10.1111/pbi.12861 PMC5978870

[CR52] Zhang L, Liu J, Cheng J, Sun Q, Zhang Y, Liu J, et al. lncRNA7 and lncRNA2 modulate cell wall defense genes to regulate cotton resistance to *Verticillium wilt*. Plant Physiol. 2022;189:264. 35134243 10.1093/plphys/kiac041 PMC9070856

[CR53] Zhang L, Ren Y, Meng F, Bao H, Xing F, Tian C. Verification of the protective effects of poplar phenolic compounds against poplar anthracnose. Phytopathology^®^. 2022;112:1943. 10.1094/PHYTO-12-21-0509-R 35578737

[CR54] Zheng W, Hu H, Lu Q, Jin P, Cai L, Hu C, et al. Genome-wide identification and characterization of long noncoding rnas involved in Chinese wheat mosaic virus infection of *Nicotiana Benthamiana*. Biology. 2021;10:232. 33802832 10.3390/biology10030232 PMC8002735

[CR55] Zhou LJ, Geng Z, Wang Y, Wang Y, Liu S, Chen C, et al. A novel transcription factor CmMYB012 inhibits flavone and anthocyanin biosynthesis in response to high temperatures in chrysanthemum. Hortic Res. 2021;8:248. 34848687 10.1038/s41438-021-00675-z PMC8633327

[CR56] Zhu QH, Stephen S, Taylor J, Helliwell CA, Wang MB. Long noncoding RNAs responsive to *Fusarium oxysporum *infection in *Arabidopsis Thaliana*. New Phytol. 2014;201:574. 24117540 10.1111/nph.12537

