# Supplementary material for: Sorbitol induces flavonoid accumulation as a secondary signal via the nanoencapsulated SPc/lncRNA809-MmNAC17 module against Alternaria alternata in Malus micromalus
Source: Mol Hortic. 2025 Jan 31;5:5. doi: 10.1186/s43897-024-00125-z (PMC11783756; doi:10.1186/s43897-024-00125-z)
Supplement: Supplementary file 7 — Supplementary Material 7: Supplemental Figure 1. Isolation and identification of the main pathogenic fungus R1 from M. micromalus. (A) The isolation process of pathogenic fungi from M. micromalus. (B) Pathogenicity identification of R1, R2, and R3. Scale bar, 1 cm. (C) Phylogenetic tree analysis of R1. R1 belongs to the genus A. alternata R1. Supplemental Figure 2. The content of sorbitol in the leaves of M. micromalus fed with exogenous sorbitol is detected. The data is the mean ± SD (n=3). *p < 0.05, two-sided Student’s t-test. Supplemental Figure 3. The GO pathways that are enriched by differentially expressed genes regulated by the pathogen R1. Group 2, H2O vs H2O+R1. Group 3, sorbitol vs sorbitol+R1. Here are the top 15 entries with p < 0.05. Supplemental Figure 4. The KEGG pathways that are enriched by differentially expressed genes. (A) The KEGG pathways of downregulated genes in Group 1 and Group 4 are shown. (B) The KEGG pathways of upregulated and downregulated genes in Group 2 and Group 3 aredisplayed respectively. Supplemental Figure 5. A heatmap with the expression level of genes related to flavonoid synthesis.Supplemental Figure 6. Family member analysis of genes related to flavonoid synthesis. Supplemental Figure 7. The concentration of catechin that inhibit the growth of pathogens is determined.The antibacterial effect (A), pathogen growth diameter (B), and spore count (C) are shown. Scale bar, 2 cm. Supplemental Figure 8. The antibacterial effects of naringenin in vitro. R1 was inoculated onto PDA medium with naringenin and cultured at 25℃ for 96 hours. The growth process (A) and spore morphology (C) of pathogen R1 are displayed in the presence or absence of naringenin. Scale bars, 4.5 cm (A) and 20 μm (C). The growth diameter (B), spore quantity (D), and spore germination (E) are also statistically analyzed. Supplemental Figure 9. The antibacterial effects of quercetin in vitro. R1 was inoculated onto PDA medium with quercetin and cultured at 25℃ f [file 43897_2024_125_MOESM7_ESM.docx]

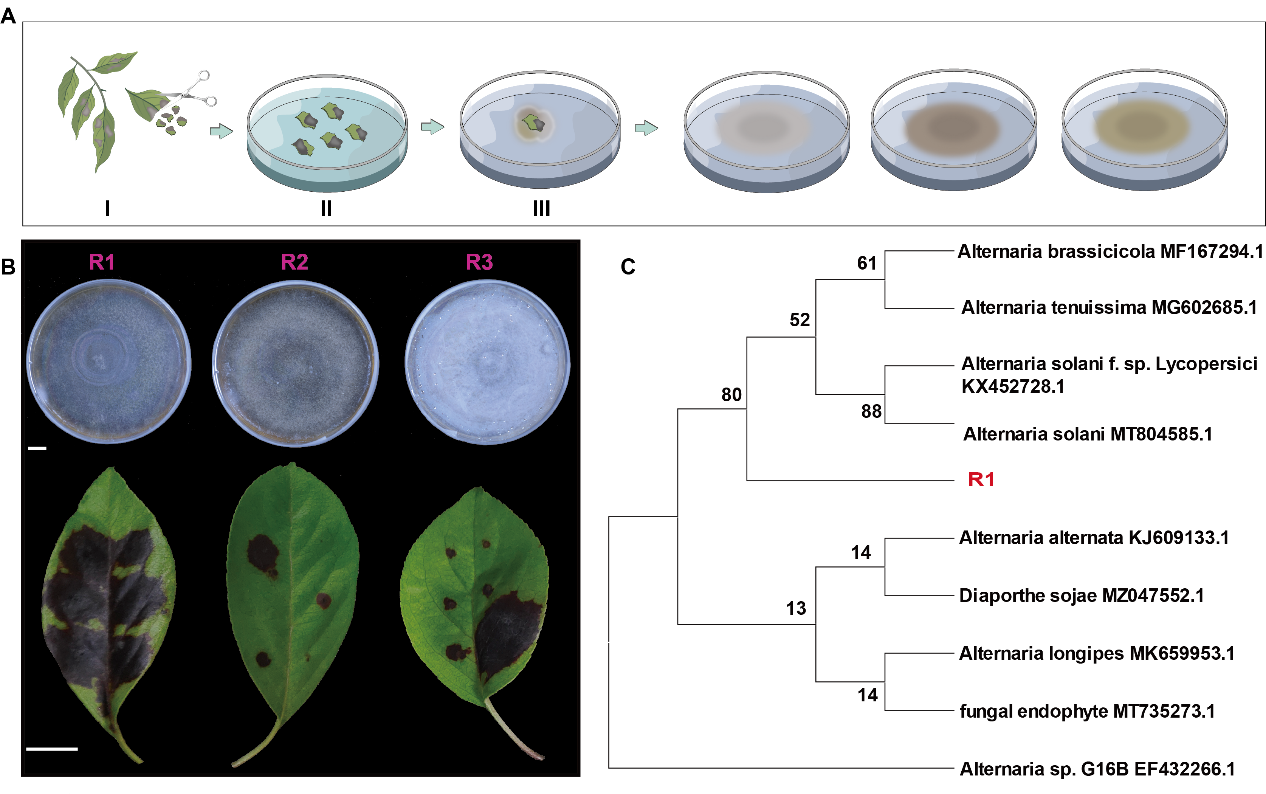


**Supplemental Figure 1 Isolation and identification of the main pathogenic fungus R1 from *M. micromalus*.** (A) The isolation process of pathogenic fungi from *M. micromalus*. (B) Pathogenicity identification of R1, R2, and R3. Scale bar, 1 cm. (C) Phylogenetic tree analysis of R1. R1 belongs to the genus *Alternaria*.


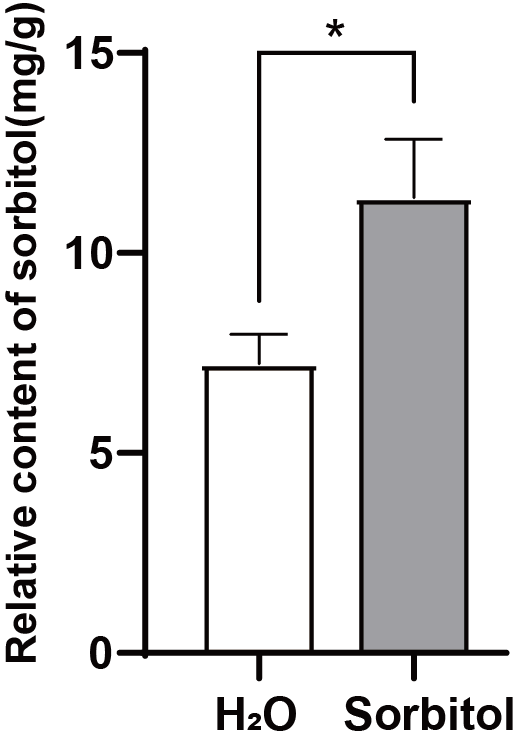


**Supplemental Figure 2 The content of sorbitol in the leaves of *M. micromalus* fed with exogenous sorbitol.** The data is the mean ± SD (n=3). *p<0.05, two-sided Student’s t-test.


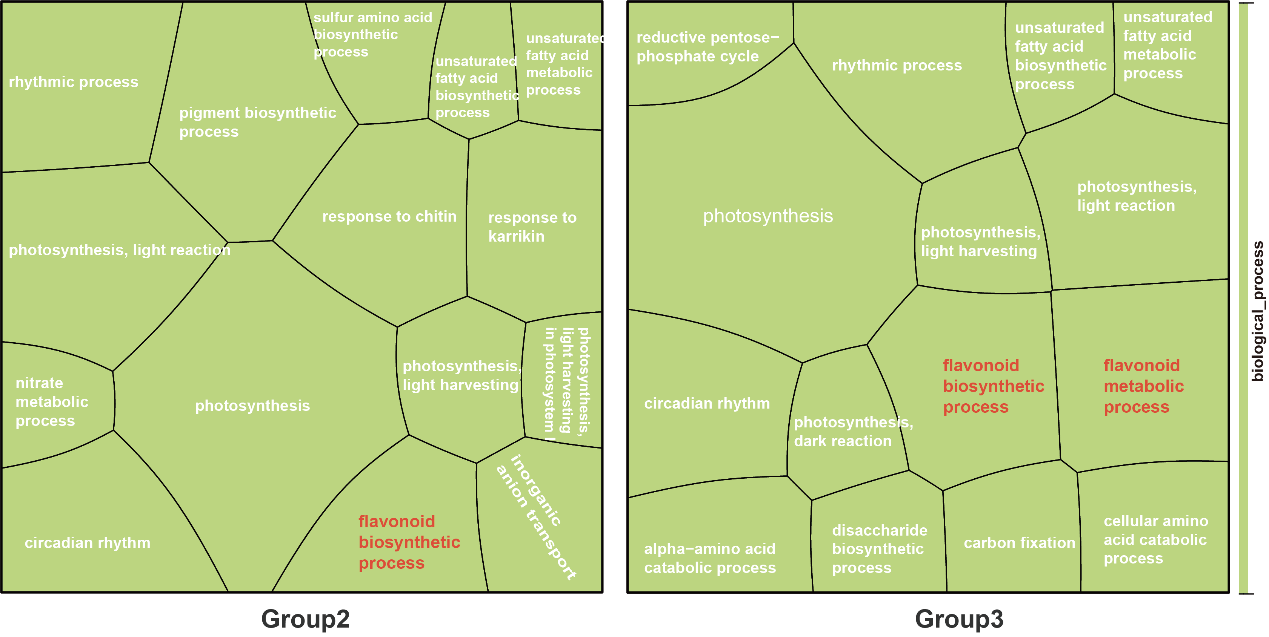


**Supplemental Figure 3 The GO pathway enriched by differentially expressed genes regulated by the pathogen R1.** Group 2, H_2_O vs H_2_O+R1. Group 3, sorbitol vs sorbitol+R1. Here are the top 15 entries with p<0.05.


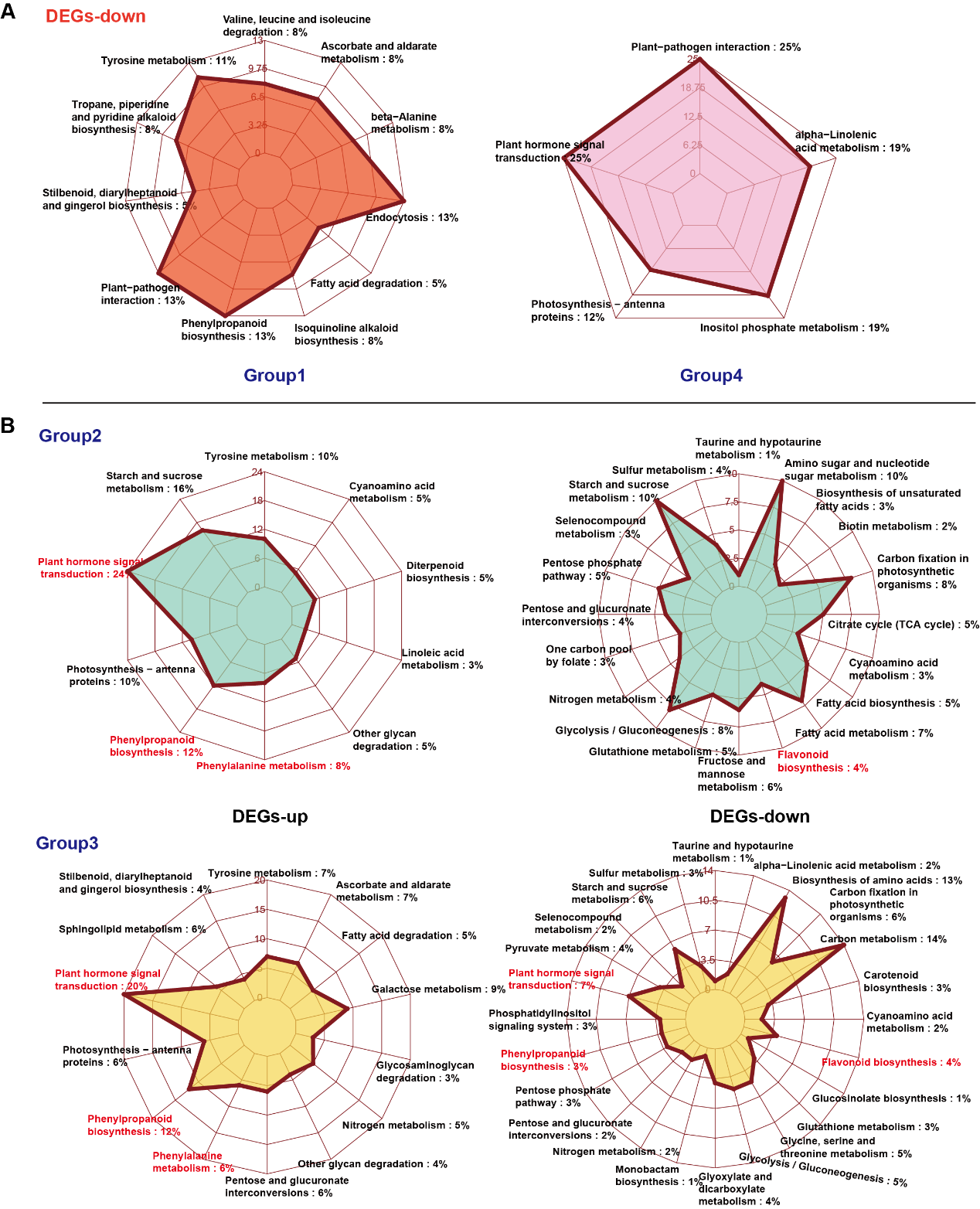


**Supplemental Figure 4 The KEGG pathway enriched by differentially expressed genes.** (A) The KEGG pathway of downregulated genes in Group 1 and Group 4 was shown. (B) The KEGG pathways of upregulated and downregulated genes are displayed respectively in Group 2 and Group 3.


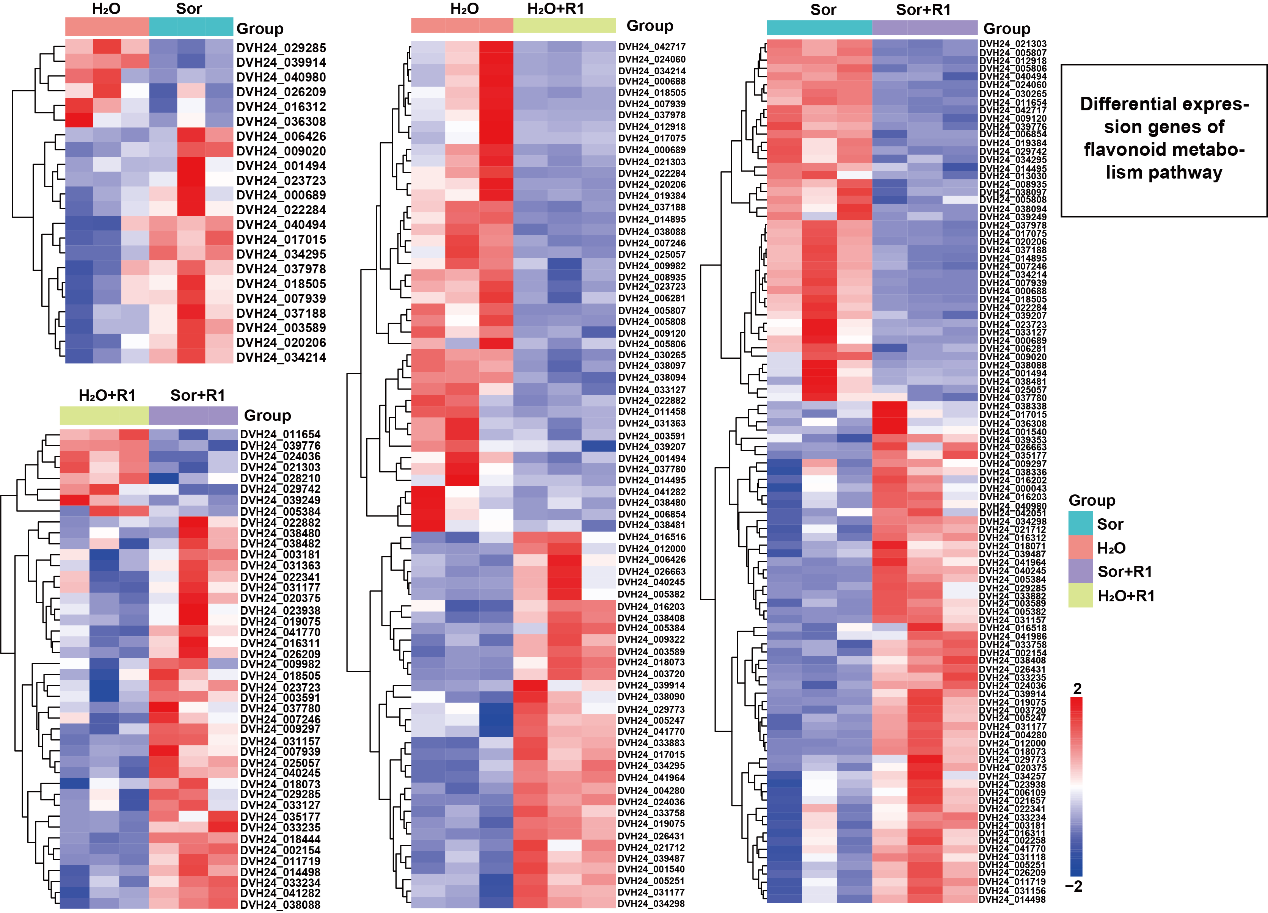


**Supplemental Figure 5 A heatmap shown the expression level of genes related to flavonoid synthesis.**


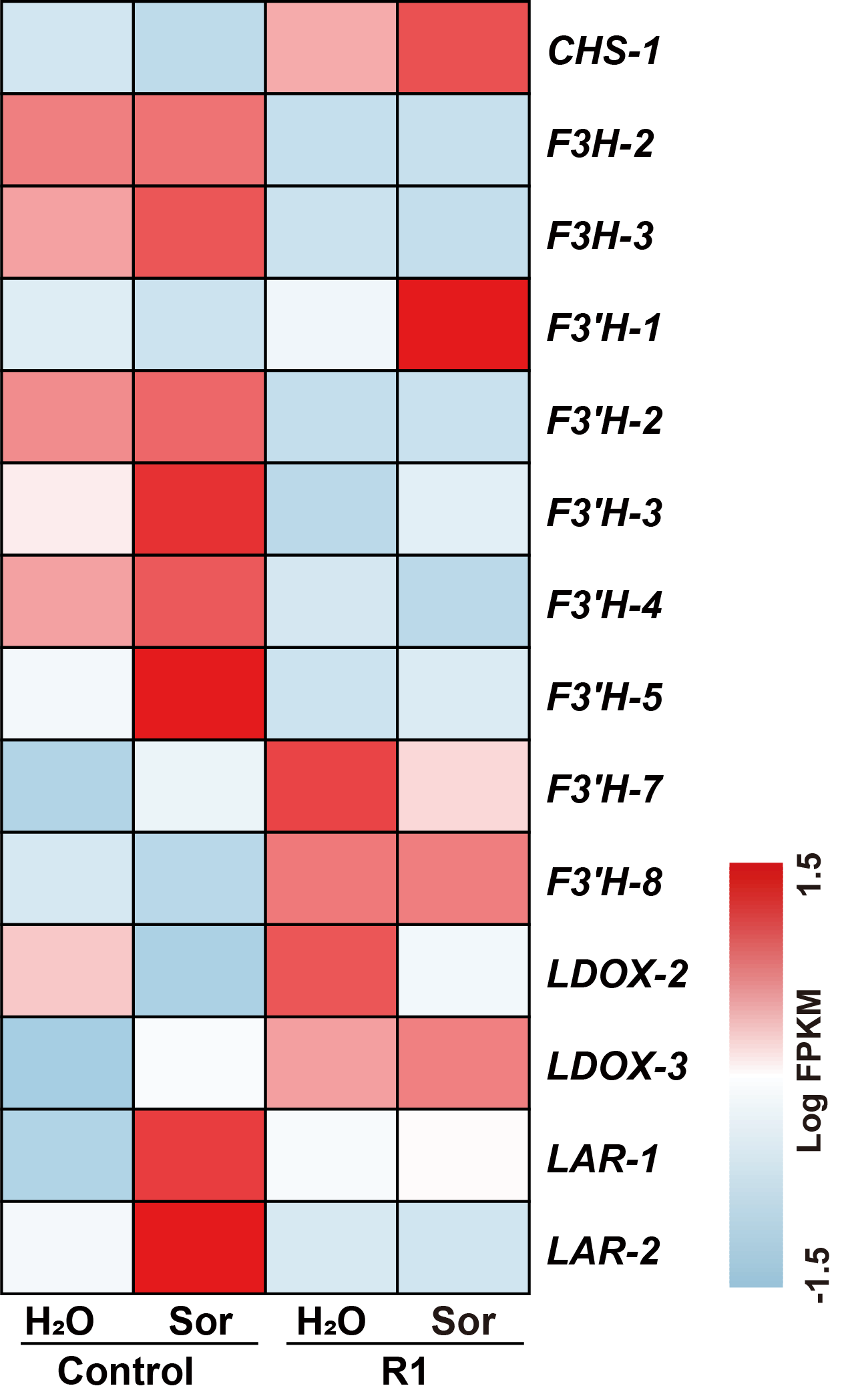


**Supplemental Figure 6 Family member analysis of genes related to flavonoid synthesis.**


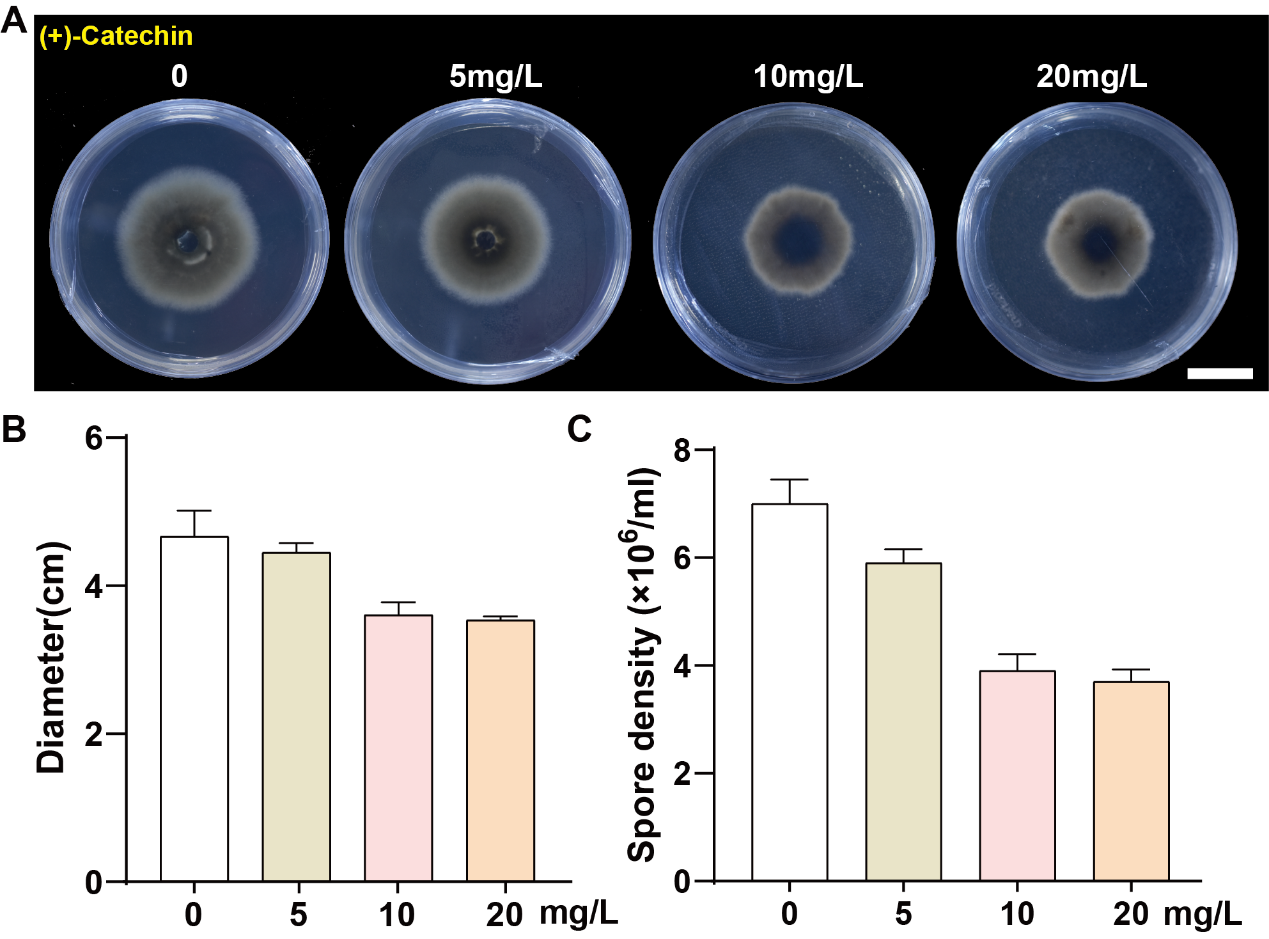


**Supplemental Figure 7 The concentration of catechins that inhibit the growth of pathogens.** The antibacterial effect (A), pathogen growth diameter (B), and spore count (C) are shown. Scale bar, 2 cm.


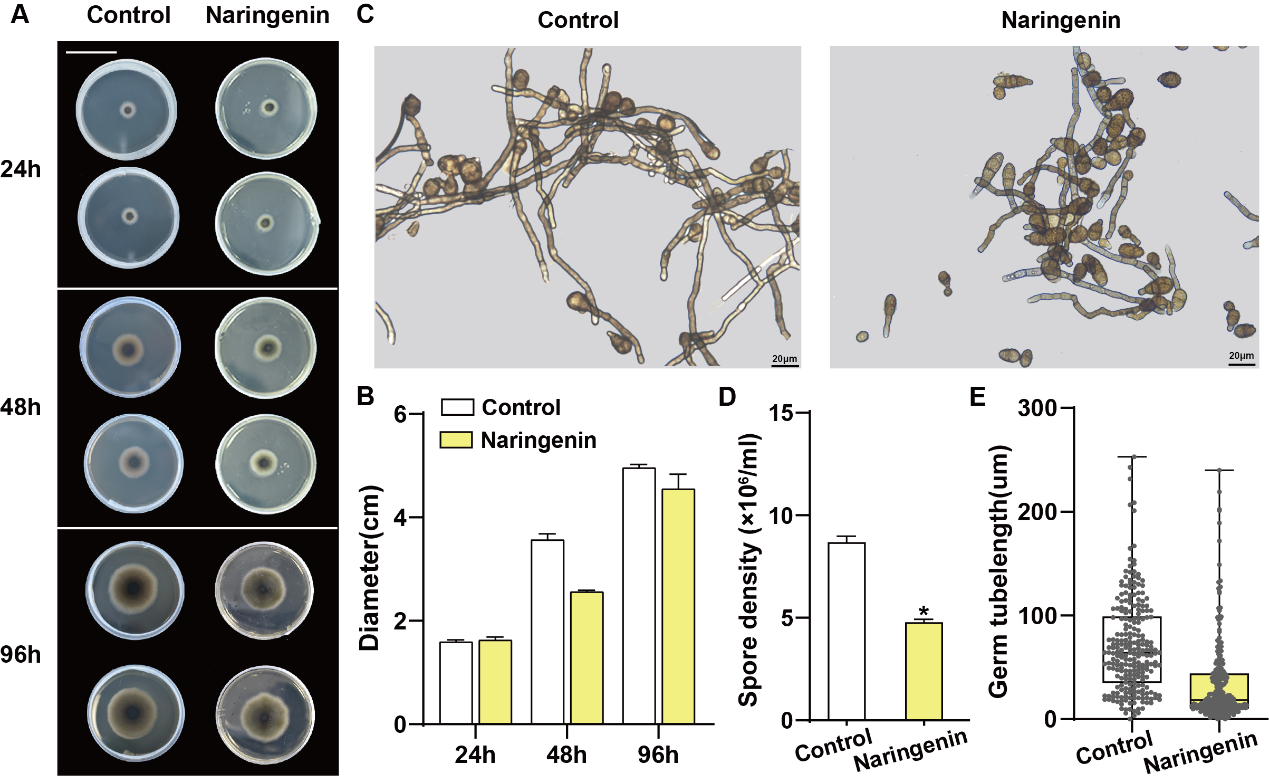


**Supplemental Figure 8 The antibacterial effects of naringenin *in vitro*.** R1 was inoculated onto PDA medium with naringenin and cultured at 25℃ for 96 hours. The growth process (A) and spore morphology (C) of pathogen R1 are displayed in the presence or absence of naringenin. Scale bars, 4.5 cm (A) and 20 μm (C). The growth diameter (B), spore quantity (D), and spore germination (E) are also statistically analyzed.


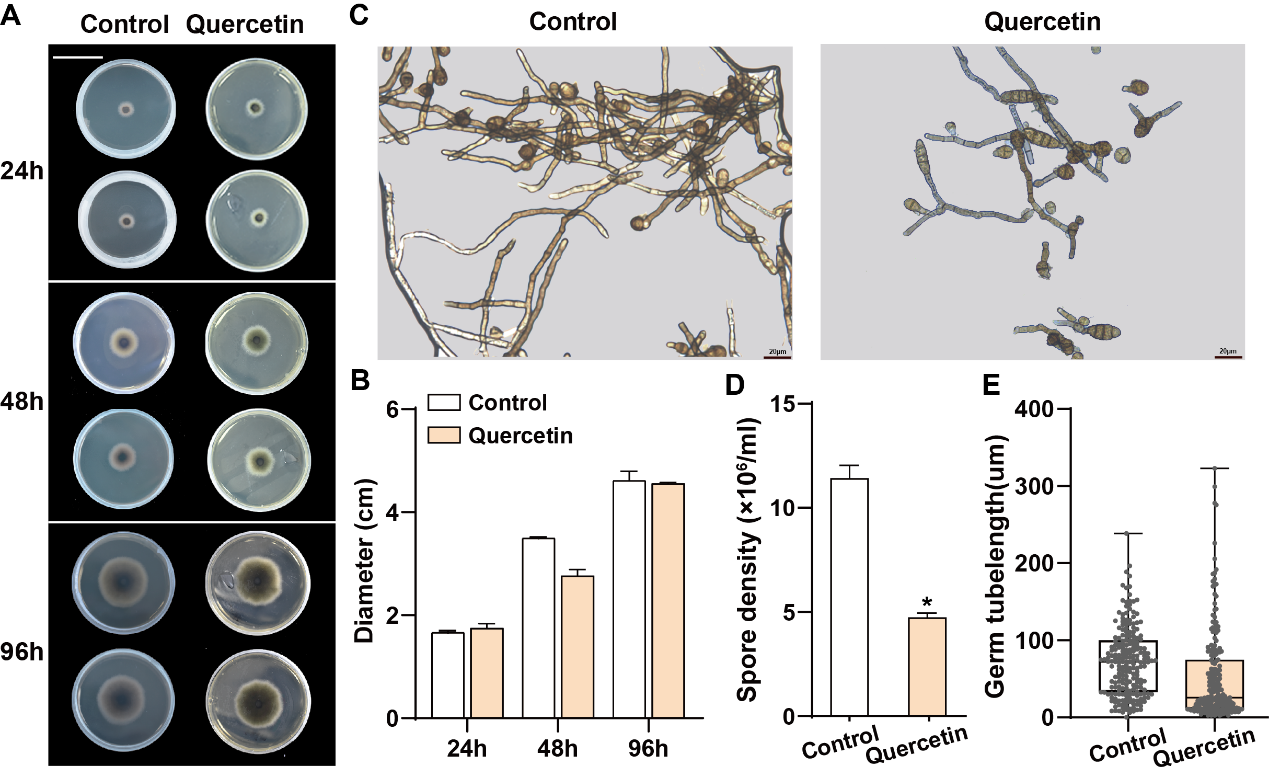


**Supplemental Figure 9 The antibacterial effects of** **quercetin *in vitro*.** R1 was inoculated onto PDA medium with quercetin and cultured at 25℃ for 96 hours. The growth process (A) and spore morphology (C) of pathogen R1 are displayed in the presence or absence of quercetin. Scale bars, 4.5 cm (A) and 20 μm (C). The growth diameter (B), spore quantity (D), and spore germination (E) are also statistically analyzed.


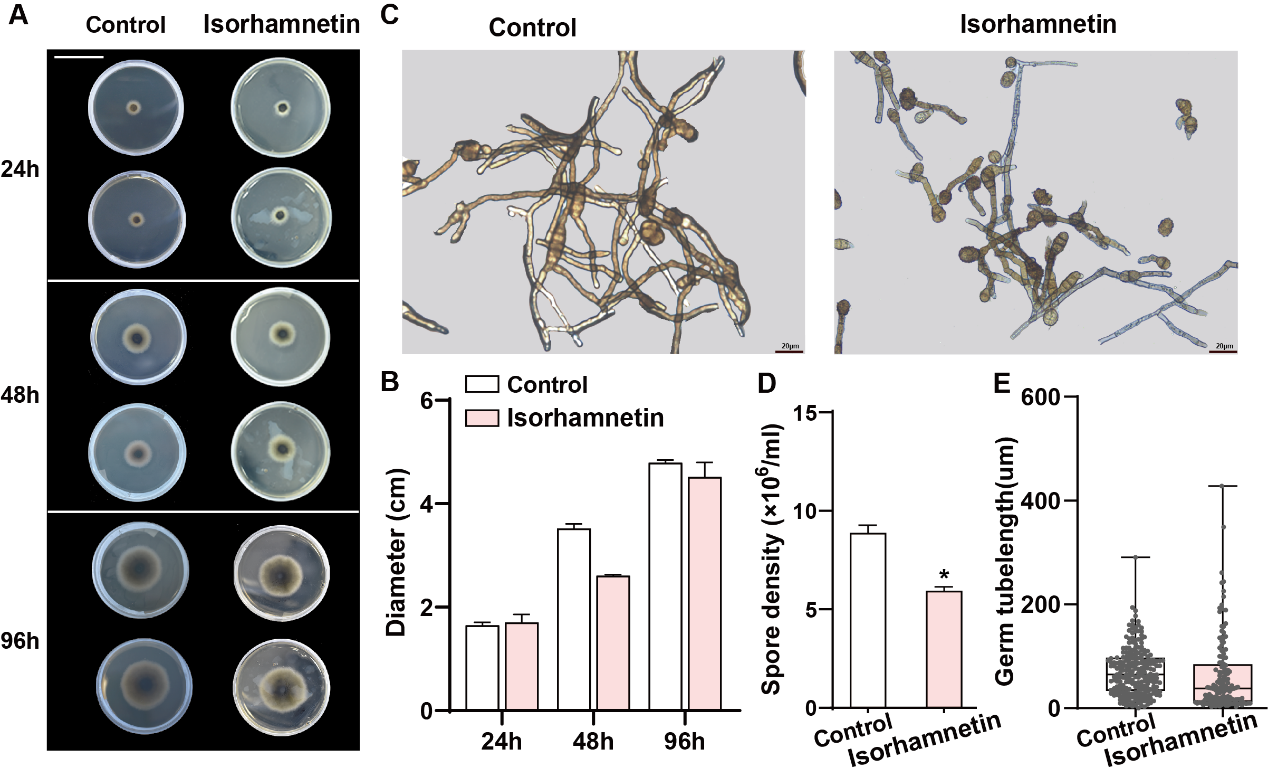


**Supplemental Figure 10 The antibacterial effects of** **isorhamnetin *in vitro*.** R1 was inoculated onto PDA medium with isorhamnetin and cultured at 25℃ for 96 hours. The growth process (A) and spore morphology (C) of pathogen R1 are displayed in the presence or absence of isorhamnetin. Scale bars, 4.5 cm (A) and 20 μm (C). The growth diameter (B), spore quantity (D), and spore germination (E) are also statistically analyzed.


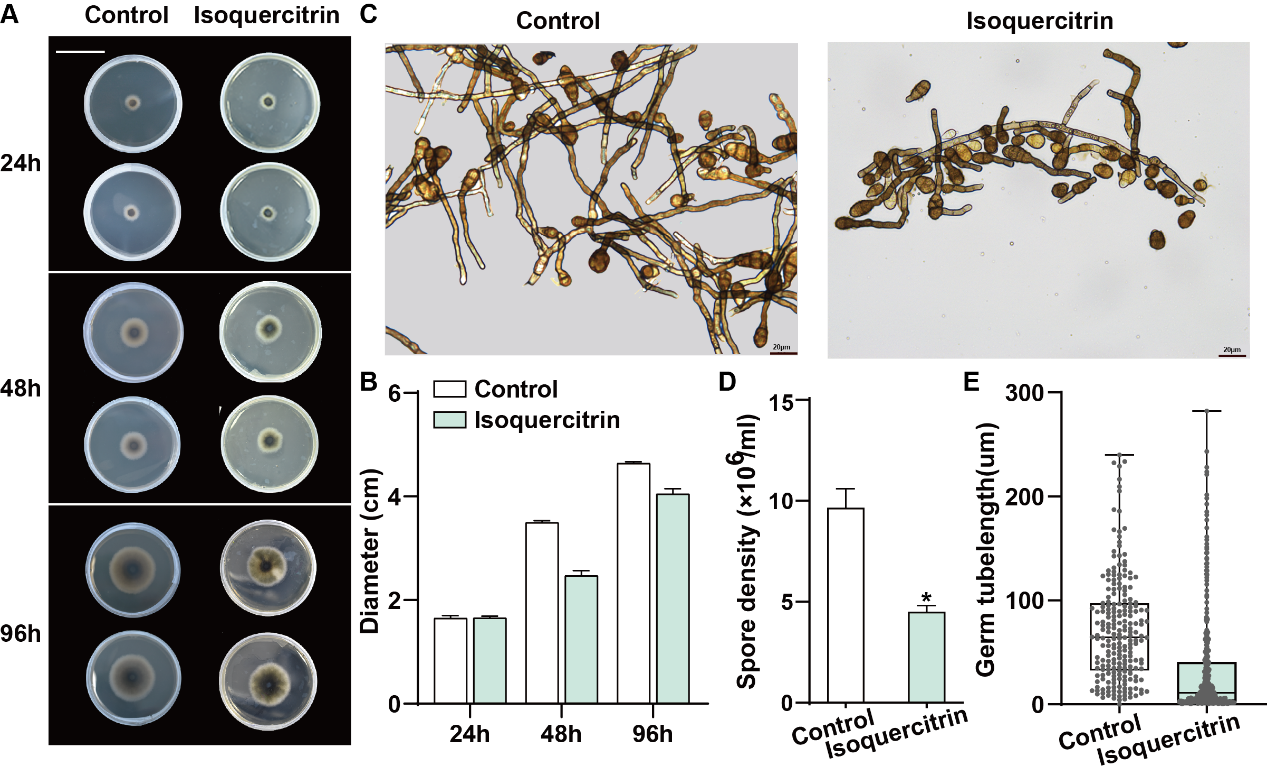


**Supplemental Figure 11 The antibacterial effects of** **isoquercitrin *in vitro*.** R1 was inoculated onto PDA medium with isoquercitrin and cultured at 25℃ for 96 hours. The growth process (A) and spore morphology (C) of pathogen R1 are displayed in the presence or absence of isoquercitrin. Scale bars, 4.5 cm (A) and 20 μm (C). The growth diameter (B), spore quantity (D), and spore germination (E) are also statistically analyzed.


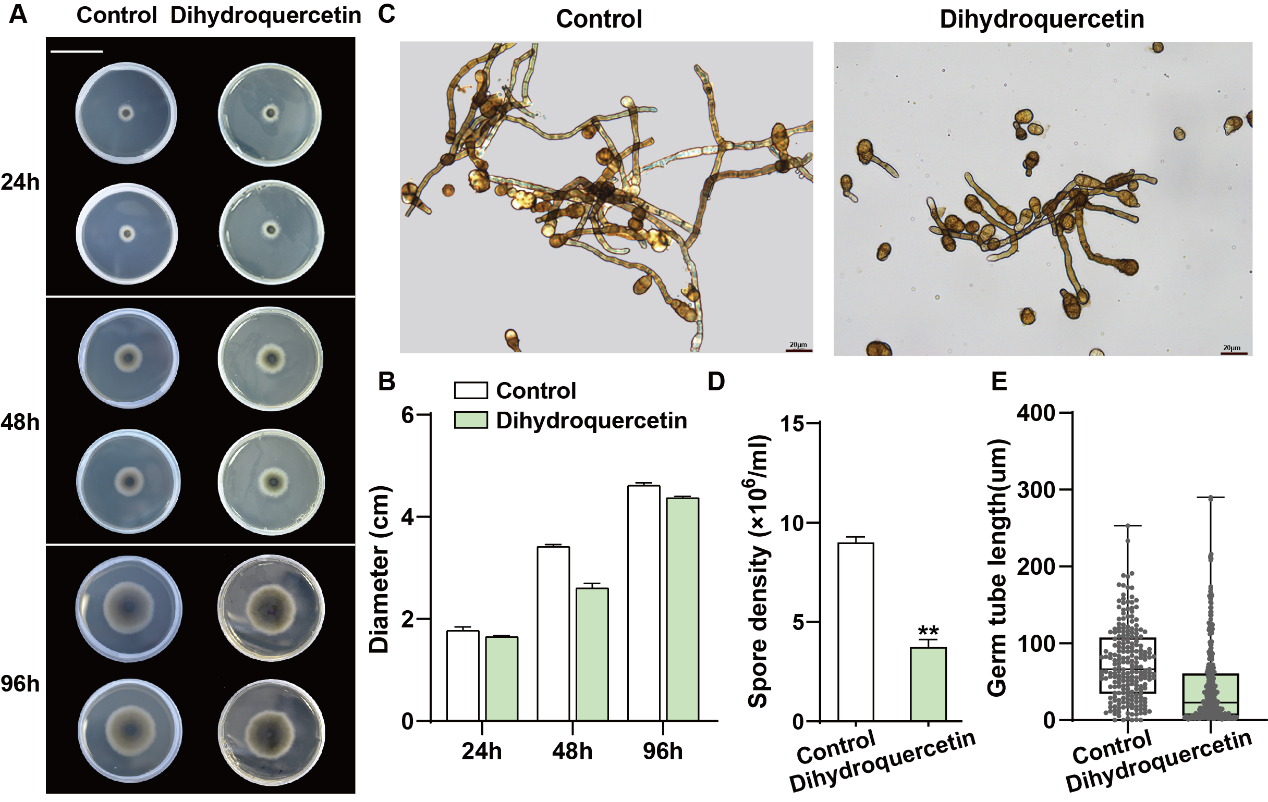


**Supplemental Figure 12 The antibacterial effects of** **dihydroquercetin *in vitro*.** R1 was inoculated onto PDA medium with dihydroquercetin and cultured at 25℃ for 96 hours. The growth process (A) and spore morphology (C) of pathogen R1 are displayed in the presence or absence of dihydroquercetin. Scale bars, 4.5 cm (A) and 20 μm (C). The growth diameter (B), spore quantity (D), and spore germination (E) are also statistically analyzed.


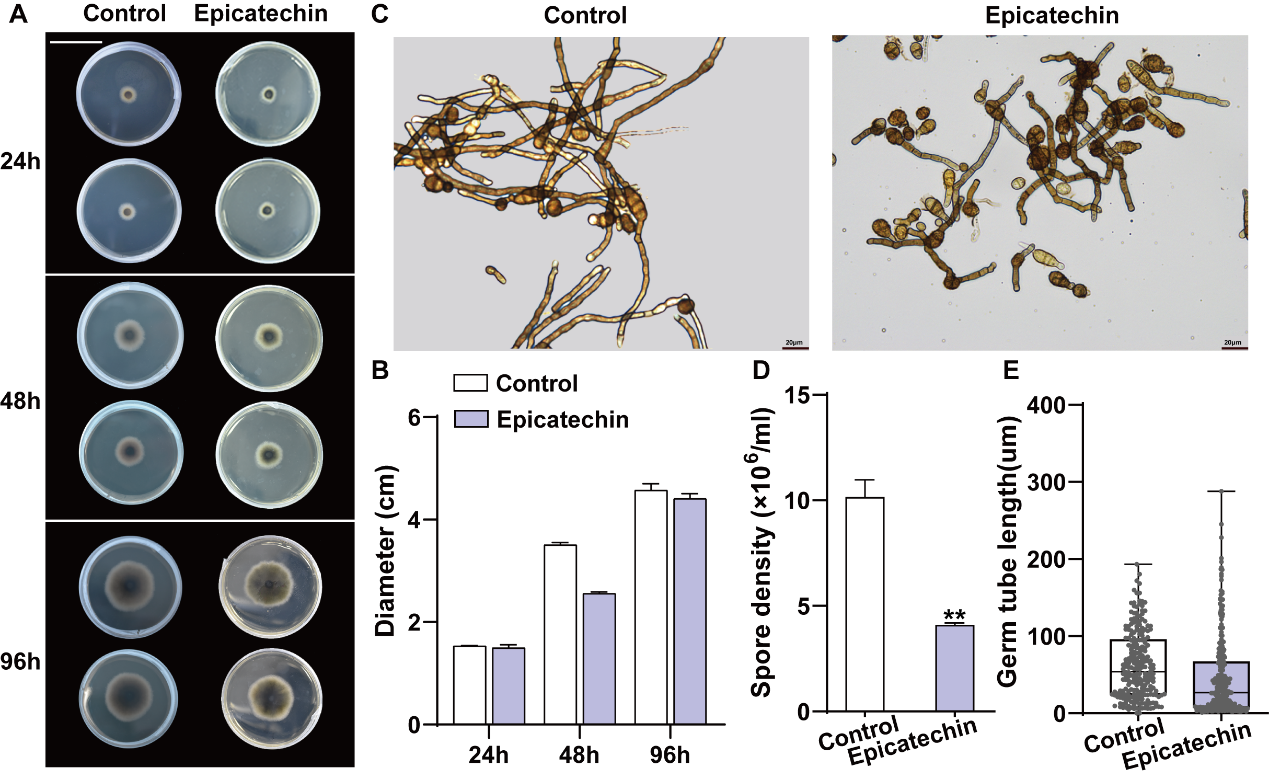


**Supplemental Figure 13 The antibacterial effects of** **epicatechin *in vitro*.** R1 was inoculated onto PDA medium with epicatechin and cultured at 25℃ for 96 hours. The growth process (A) and spore morphology (C) of pathogen R1 are displayed in the presence or absence of epicatechin. Scale bars, 4.5 cm (A) and 20 μm (C). The growth diameter (B), spore quantity (D), and spore germination (E) are also statistically analyzed.


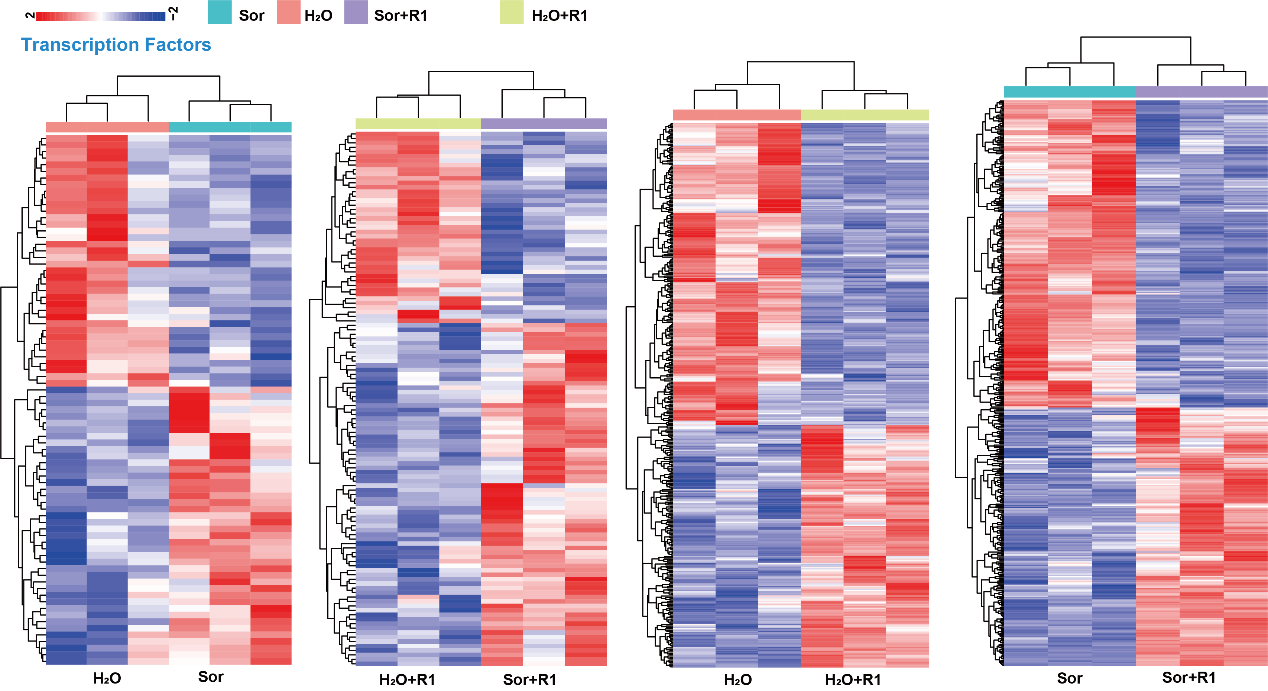


**Supplemental Figure 14 The expression levels of transcription factors are shown by HeatMap.**


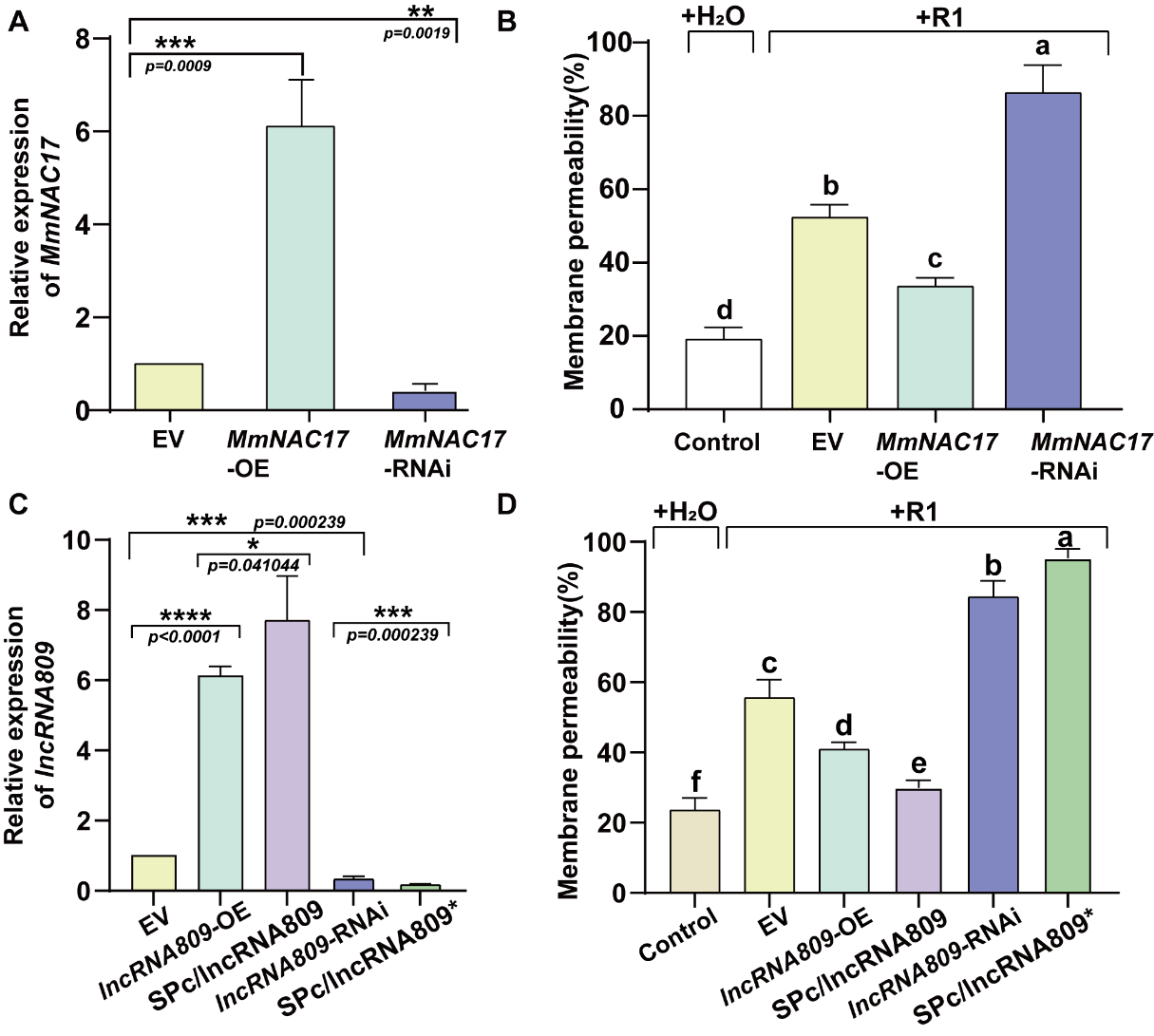


**Supplemental Figure 15 Expression levels of MmNAC17 and lncRNA809 and cell membrane permeability of leaves under pathogen R1 infection.** The relative expression levels of MmNAC17 and lncRNA809 (A, C). Transcripts were quantified by RT-qPCR. The data is the mean ± SD (n=3). **p<0.01, ***p<0.001, two-sided Student’s t-test. The cell membrane permeability of leaves (B, D). ANOVA, Tukey’s multiple comparisons, P<0.05, different letters indicate significant differences.


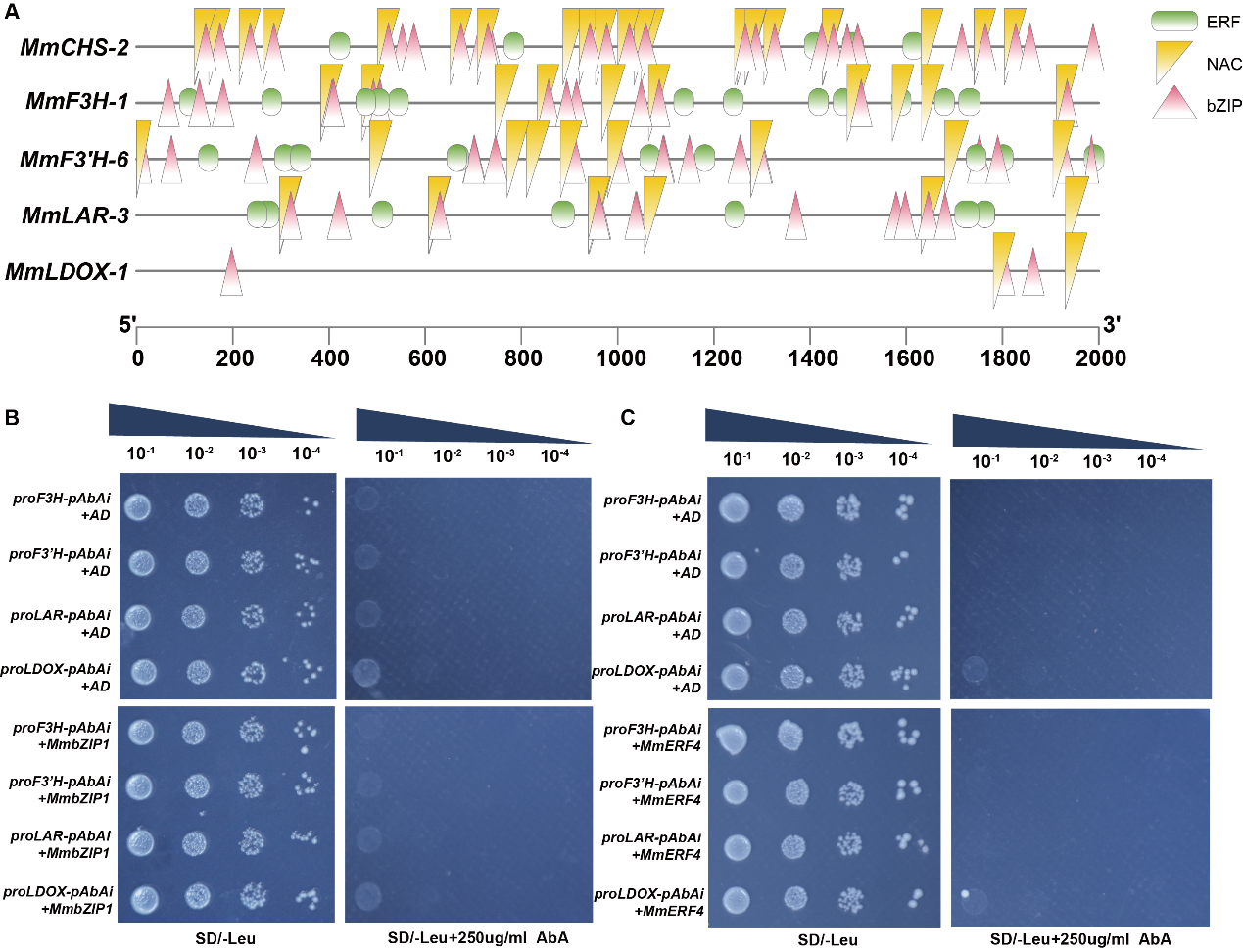


**Supplemental Figure 16 Prediction of promoter binding elements for flavonoid synthesis related genes and Y1H assays.** (A) The transcription factor binding sites predicted using the plantpan3.0 database are displayed. (B) Y1H assay showing no interaction between transcription factors MmbZIP1 and MmERF4 and promoter fragments of flavonoid synthesis related genes. Empty pGADT7 vectors were used as a negative control.


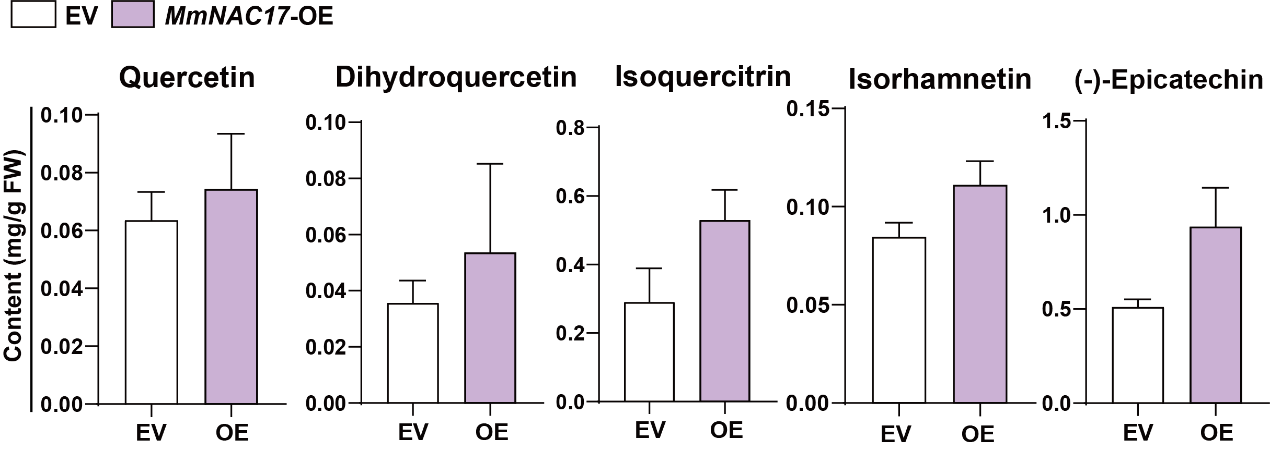


**Supplemental Figure 17 The effect of MmNAC17 on other flavonoids.**


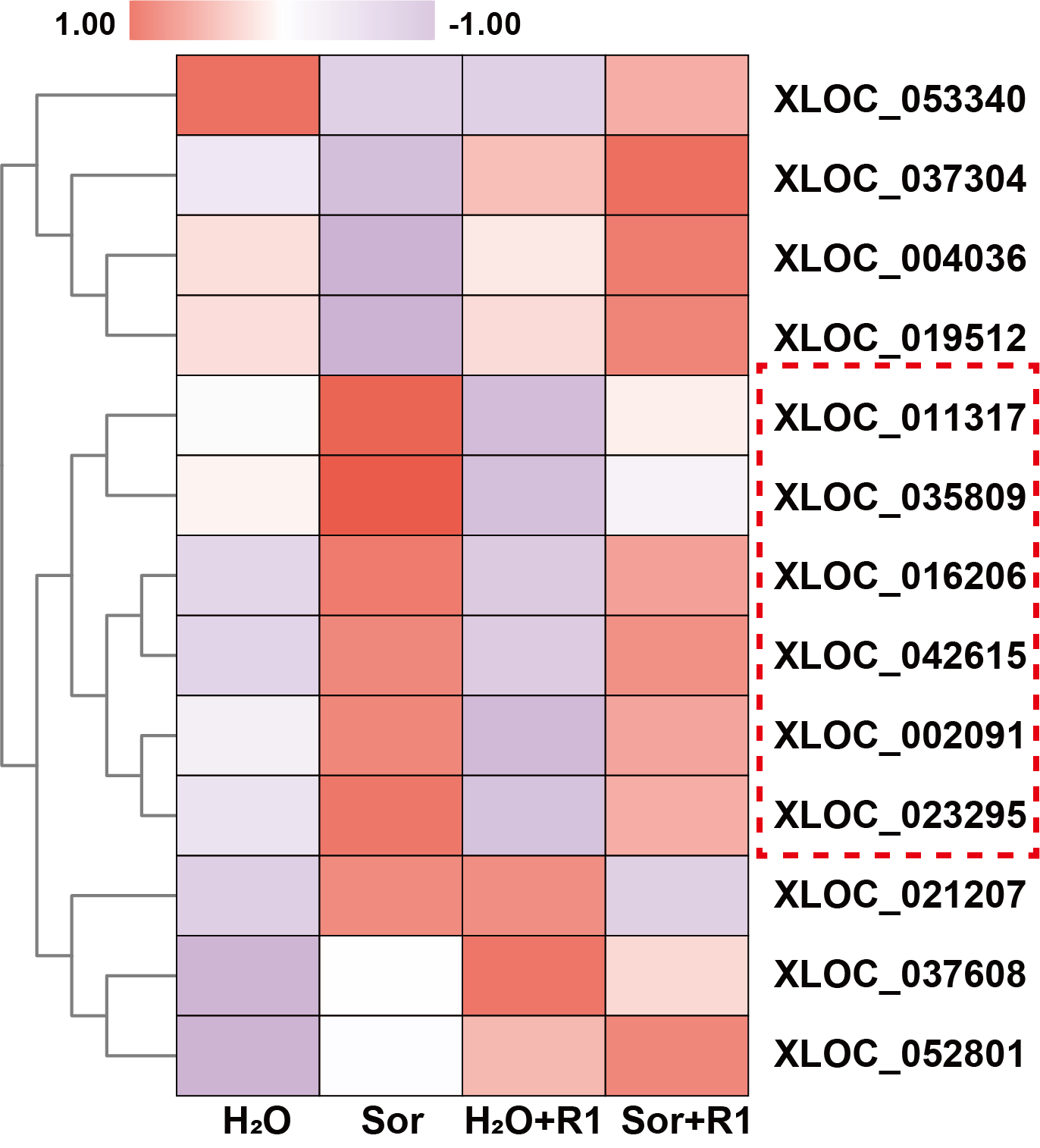


**Supplemental Figure 18 Heat map of differentially expressed lncRNAs.**

**
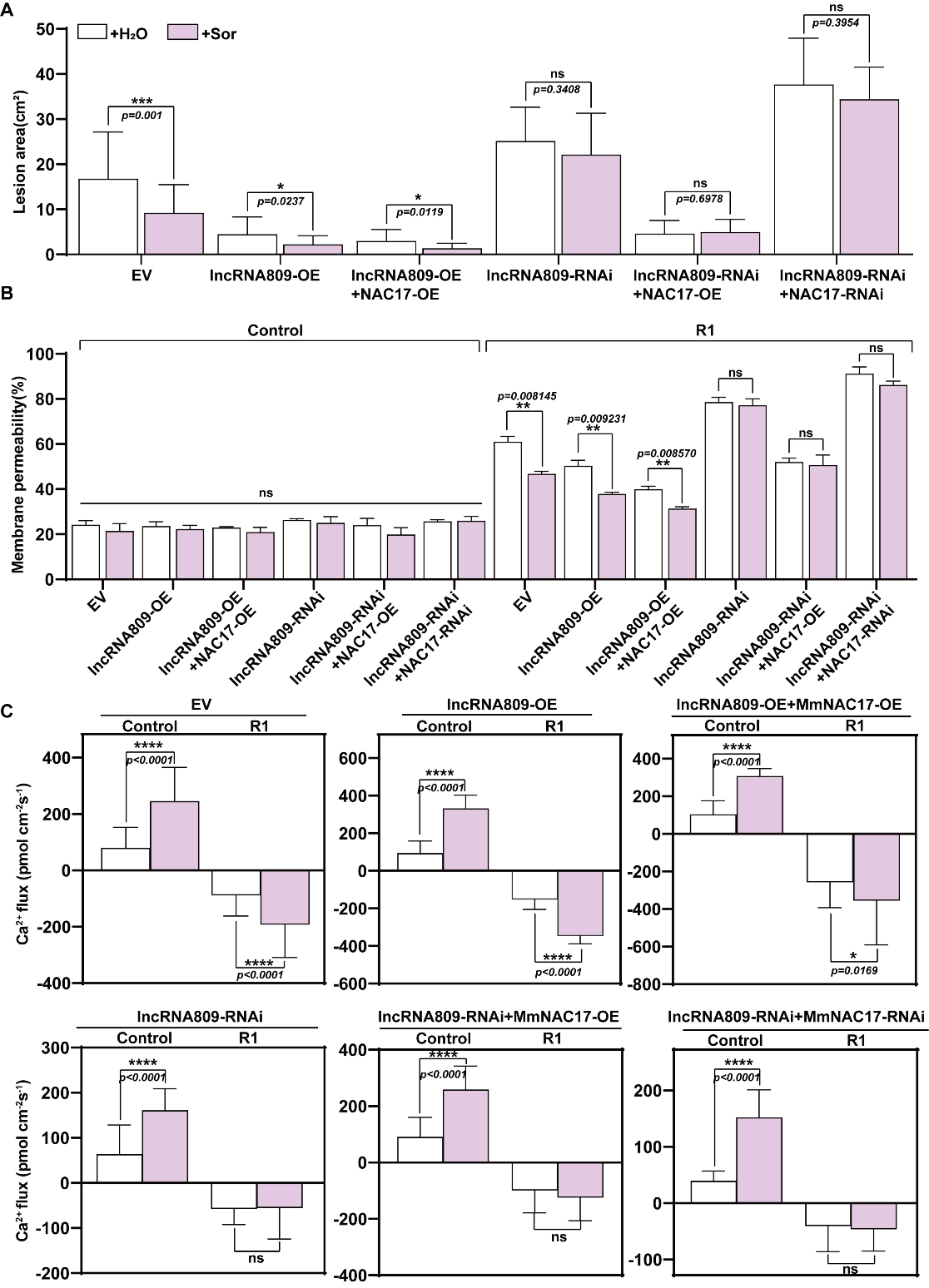
**

**Supplemental Figure 19 The lesion area, cell membrane permeability, and calcium ion flux of transgenic leaves under different backgrounds.** The data is the mean ± SD (n=3). *P<0.05, **p<0.01, ***p<0.001, ****p<0.0001, ns, not significant, two-sided Student’s t-test.
